# Immune-evasive beta cells in type 1 diabetes: innovations in genetic engineering, biomaterials, and computational modeling

**DOI:** 10.3389/fimmu.2025.1618086

**Published:** 2025-08-19

**Authors:** Ismail Can Karaoglu, Doğukan Duymaz, Mudassir M. Rashid, Seda Kizilel

**Affiliations:** ^1^ Chemical and Biological Engineering, Koc University, Istanbul, Türkiye; ^2^ Department of Chemical and Biological Engineering, Illinois Institute of Technology, Chicago, IL, United States

**Keywords:** type 1 diabetes, beta cell, genetic engineering, biomaterials, machine learning

## Abstract

Type 1 diabetes (T1D) is characterized by the autoimmune destruction of pancreatic beta cells, resulting in lifelong insulin therapy that falls short of a true cure. Beta cell replacement therapies hold immense potential to restore natural insulin production, but they face significant hurdles such as immune rejection, limited donor availability, and long-term graft survival. In this review, we explore cutting-edge advances in genetic engineering, biomaterials, and machine learning approaches designed to overcome these barriers and enhance the clinical applicability of beta cell therapies. We highlight recent innovations in genetic editing techniques, particularly CRISPR/Cas9-based strategies, aimed at generating hypoimmune beta cells capable of evading immune detection. Additionally, we discuss novel biomaterial encapsulation systems, engineered at nano-, micro-, and macro-scales, which provide physical and biochemical protection, promote graft integration, and survival. We mention that recent advances in machine learning and computational modeling also play a crucial role in optimizing therapeutic outcomes, predicting clinical responses, and facilitating personalized treatment approaches. We also critically evaluate ongoing clinical trials, providing insights into the current translational landscape and highlighting both successes and remaining challenges. Finally, we propose future directions, emphasizing integrated approaches that combine genetic, biomaterial, and computational innovations to achieve durable, scalable, and immunologically tolerant beta cell replacement therapies for T1D.

## 1 Introduction

### 1.1 T1D immunology

Type 1 diabetes (T1D) is an autoimmune disease where the insulin secreting beta cells are destroyed by immune cells, leading to increased blood glucose levels. Beta cells are located in the Islets of Langerhans within the pancreas and are responsible for the production of a vital hormone called insulin. Due to the lack of insulin, T1D patients experience hyperglycemia, which can cause severe complications such as heart disease, stroke, nerve damage, and kidney failure. T1D develops in childhood or adolescence but can also occur in adults, where the disease onset and progression are triggered by either environmental or immunological events (1).

**Figure 1:**
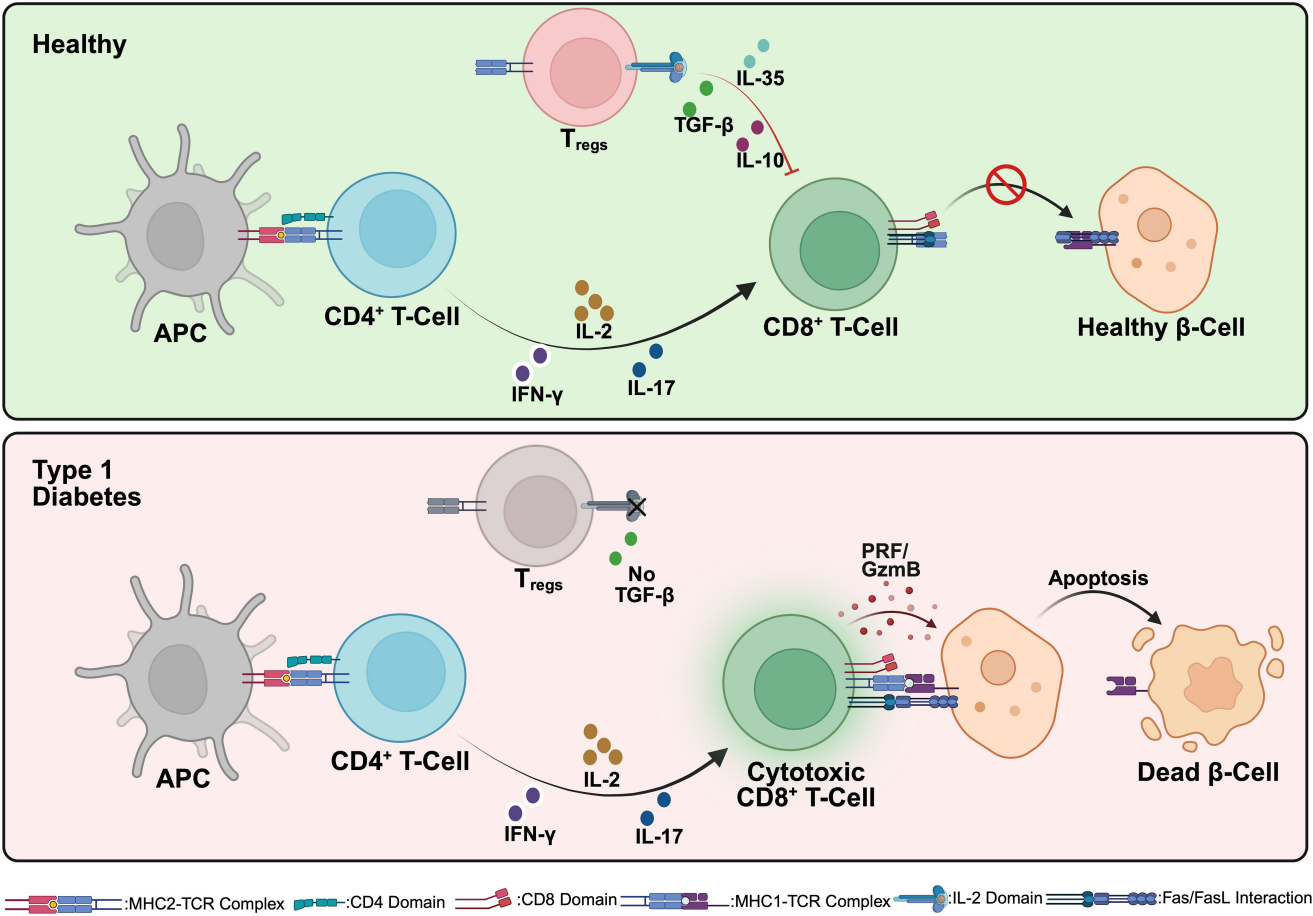
Immunological interactions in healthy individuals and Type 1 Diabetes (T1D). In a healthy state (top), antigen-presenting cells (APCs) interact with CD4^+^ T cells, resulting in the differentiation and expansion of regulatory T cells (T_regs_). These T_regs_ secrete immunosuppressive cytokines (IL-10 and TGF-β), maintaining peripheral immune tolerance by inhibiting the activation of autoreactive CD8^+^ cytotoxic T cells. Consequently, pancreatic beta cells remain healthy and functional. In contrast, during T1D pathogenesis (bottom), APCs activate autoreactive CD4^+^ T cells, which differentiate into pro-inflammatory subsets (such as Th1 and Th17). These cells produce inflammatory cytokines, including IFN-γ and IL-17, driving the activation and proliferation of autoreactive cytotoxic CD8^+^ T cells. The activated CD8^+^ T cells then induce beta cell apoptosis via direct mechanisms (perforin/granzyme and Fas/Fas ligand pathways), resulting in progressive beta cell destruction and the clinical manifestation of T1D. Created in https://BioRender.com.

T1D has a strong genetic component, with susceptibility largely linked to HLA class II genes (HLA-DR, HLA-DQ, and HLA-DP). Certain haplotypes, such as HLA-DR3/DR4, are associated with a higher risk, whereas other variants may confer protection (2). Additionally, non-HLA genes like INS (insulin gene), PTPN22, CTLA-4, and IL2RA play crucial roles in immune regulation and T1D susceptibility (3). In healthy individuals, central tolerance mechanisms in the thymus eliminate self-reactive T cells through negative selection. However, in genetically susceptible individuals, autoreactive CD4^+^ T helper cells (Th1 and Th17 subsets) escape deletion and become activated in peripheral lymphoid tissues. A key event in T1D pathogenesis is the presentation of beta cell antigens by antigen-presenting cells (APCs) (4). Dendritic cells and macrophages engulf beta cell-derived proteins (e.g., insulin, GAD65, IA-2, ZnT8) and present them to naive T cells via HLA class II molecules. This leads to the activation of: (i) CD4^+^ T cells, which orchestrate immune responses by secreting proinflammatory cytokines such as IFN-γ, IL-2, and IL-17; and (ii) CD8^+^ cytotoxic T lymphocytes (CTLs), which directly mediate beta cell destruction through the perforin/granzyme and Fas-FasL pathways. Regulatory T cells (T_regs_), which normally suppress autoimmunity, are dysfunctional in T1D, allowing excessive immune activation (**Figure 1**). Additionally, B cells contribute to autoimmunity by producing islet autoantibodies against insulin, GAD65, and IA-2, which serve as biomarkers for disease progression (5).

The pancreatic islets in T1D are infiltrated by immune cells in a process called insulitis. This inflammatory environment is enriched with TNF-α, IFN-γ, and IL-1β, which impair beta cell function and enhance apoptosis. In parallel, beta cells under attack initiate stress responses and secrete chemokines (e.g., CXCL10, CCL5) that further recruit immune cells, amplifying the autoimmune loop. Beta cell destruction occurs through multiple mechanisms: (i) direct killing by CD8^+^ T cells via granzyme B/perforin-mediated cytotoxicity, (ii) Fas - Fas ligand (FasL) signaling, where beta cells expressing Fas undergo apoptosis upon interaction with FasL-expressing T cells, (iii) cytokine-induced dysfunction, as TNF-α, IL-1β, and IFN-γ activate endoplasmic reticulum (ER) stress and the JAK-STAT and NF-κB pathways, leading to metabolic stress and apoptosis (6). The intricate interplay between T cells, B cells, APCs, and beta cells emphasizes the challenges in developing therapies to halt or reverse the disease. Understanding these mechanisms is crucial for designing targeted interventions, including immune modulation, beta cell replacement, and genetic engineering approaches to restore glucose homeostasis.

### 1.2 Current treatments in T1D

The clinical management of T1D has traditionally relied on exogenous insulin therapy, a lifesaving yet ultimately non-curative intervention. Since insulin’s discovery in 1921, therapeutic approaches have significantly evolved from multiple daily injections (MDI) to continuous subcutaneous insulin infusion (CSII) via insulin pumps. Concurrent advancements in continuous glucose monitoring (CGM) technologies have facilitated tighter glycemic control, thereby reducing long-term complications (7).

More recently, the integration of CGM with closed-loop insulin delivery through control algorithms resulted in artificial pancreas systems, also known as automated insulin delivery systems, that have considerably improved glycemic regulation and patient quality of life. These systems employ adaptive algorithms capable of dynamically modulating insulin administration in response to real-time glucose concentration fluctuations. Despite their success in minimizing glycemic variability, artificial pancreas systems are unable to replicate the precise, glucose-responsive insulin secretion exhibited by endogenous beta cells. Furthermore, the persistent psychological and logistical burdens associated with the use of automated insulin delivery systems and devices, alongside continuous risks of hypoglycemia and hyperglycemia, highlight the inherent limitations of current insulin-based strategies. Ultimately, insulin therapy does not address the autoimmune pathogenesis underlying T1D, highlighting the necessity for curative approaches beyond exogenous insulin for glycemic management (8). Moreover, these technological solutions face inherent limitations, including the frequent requirements for user inputs for meals and physical activities, risk of hypo-/hyperglycemia, burden of device maintenance, and a fundamental inability to replicate the precise, real-time, glucose-responsive insulin secretion of endogenous beta cells. Despite substantial technological advances, insulin therapy fails to address the underlying autoimmune pathogenesis of T1D.

For patients with severe glycemic instability or hypoglycemia unawareness, cellular replacement therapies such as pancreas or islet transplantation offer a more physiological and persistent solution. Whole-pancreas transplantation is a surgical procedure where a deceased donor’s pancreas is transplanted into a recipient with T1D. This approach can achieve insulin independence and improve the quality of life by eliminating hypo-/hyperglycemic episodes, reducing the need for insulin injections and blood glucose monitoring, and offering flexibility in diet. In 1967, William Kelly performed the first successful simultaneous pancreas-kidney transplant on a 28-year-old woman with T1D and renal disease (9). The recipient survived for four and a half months post-transplant, marking a significant milestone in transplantation history. Boggi et al. transplanted whole pancreas grafts into 66 T1D patients and followed them for 10 years to assess long-term outcomes (10). The authors reported a 92.4% patient survival rate, with 57.4% achieving insulin independence and 3.2% requiring minimal insulin supplementation, suggesting that pancreas transplantation effectively restores blood glucose control in diabetic patients.

**Figure 2:**
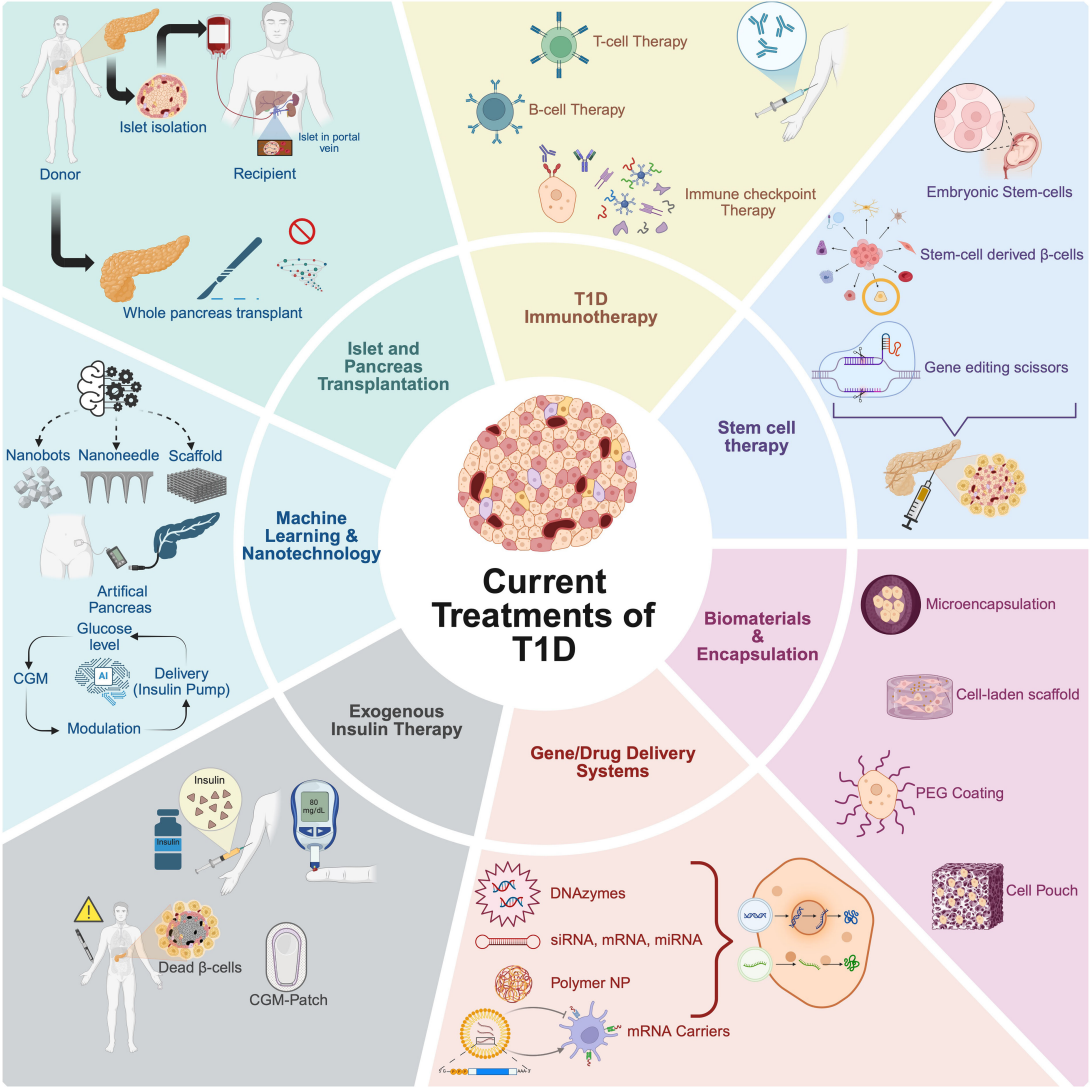
Overview of current treatments and emerging strategies for type 1 diabetes (T1D). The schematic summarizes current treatments and innovative research approaches under investigation for T1D therapy. Established clinical interventions include exogenous insulin therapy and islet or whole pancreas transplantation. Innovative strategies aiming at overcoming limitations such as immune rejection and long-term insulin dependence involve stem cell-derived beta cell therapy, utilizing pluripotent stem cells (embryonic or induced pluripotent stem cells) and genetic engineering techniques; biomaterial encapsulation and microencapsulation technologies, providing physical or biological barriers to immune attack; and advanced gene and drug delivery systems, including DNA/RNA delivery, polymeric nanoparticles (NP), and mRNA carriers, enhancing targeted and controlled therapeutic delivery. Emerging immunotherapeutic approaches include immune checkpoint modulation and T cell-based therapies to re-establish immune tolerance. Additionally, machine learning and nanotechnology applications facilitate continuous glucose monitoring, development of closed-loop artificial pancreas systems, and improved insulin delivery methods, paving the way toward personalized and precision medicine approaches in T1D treatment. Created in https://BioRender.com.

In addition to whole pancreas transplantation, The Edmonton Protocol, introduced in 2000, involves transplanting islets from cadaveric donors into patients with T1D (11). In this procedure, islets are isolated from a deceased donor’s pancreas through enzymatic and mechanical digestion, followed by density gradient purification. The islets are then infused into the recipient’s liver via the portal vein. The transplanted islets reside in the liver’s small blood vessels and begin to produce insulin. Islet transplantation is a less invasive procedure than whole-pancreas transplantation and can be performed with minimal risk using a needle under local anesthetic. Data from 571 patients between 1999 and 2010 showed that approximately 60% achieved insulin independence during the first-year post-transplant (12). However, long-term insulin independence declined over time, with most recipients eventually resuming insulin therapy, highlighting challenges in sustaining islet graft function. Marfil-Garza et al. transplanted islets into 255 T1D patients, reporting 90% survival and 70% sustained graft function over a median 7.4-year follow-up (13). While 79% achieved insulin independence, only 8% remained insulin-free at 20 years. Factors such as the use of anti-inflammatory agents (e.g., anakinra and etanercept), higher BETA-2 scores, and optimized patient selection were associated with improved outcomes. Nonetheless, widespread implementation is hindered by several major problems. One of the primary concerns is immune rejection, as transplanted beta cells are recognized as foreign by the recipient’s immune system, necessitating lifelong immunosuppression. While this prevents rejection, it also increases the risk of infections and organ toxicity (14). Additionally, limited donor availability remains a major bottleneck, with the severe shortage of cadaveric donor pancreases restricting access to these therapies. Even in cases where transplantation is successful, beta cell longevity poses another challenge, as islets often experience inflammation, immune attack, and metabolic stress, leading to functional decline and the need for repeated infusions (15). These limitations highlight the urgent need for alternative strategies that can restore beta cell function while mitigating immune rejection. This ongoing challenge has catalyzed the development of multiple innovative therapeutic strategies ranging from current exogenous insulin delivery and pancreatic and islet transplantation systems to various immune-targeting therapies, stem cell–based beta cell replacement, biomaterials-enabled encapsulation, gene and drug delivery technologies, and advanced machine learning (ML) guided platforms, each aimed at restoring immune-protected and physiologically functional insulin secretion. These approaches are schematically summarized in **Figure 2**.

In response to the challenges posed by immune rejection and donor scarcity, the field has shifted toward advanced biomaterials that aim to provide immune protection without systemic immunosuppression. Encapsulation technologies seek to create a semi-permeable barrier surrounding the transplanted islets or stem-cell–derived beta cells, permitting nutrient and insulin exchange while excluding immune cells and antibodies. Microencapsulation strategies, such as alginate-based hydrogel spheres, have shown safety and transient efficacy in early clinical studies. However, pericapsular fibrotic overgrowth and impaired oxygen diffusion have limited long-term graft survival (16). To overcome these barriers, next-generation materials now incorporate protein-repellent polymers like PEG or zwitterions, as well as surface-bound immunoregulatory ligands such as PD-L1 and FasL (17). Macroencapsulation platforms such as ViaCyte’s Encaptra device and the Sernova Cell Pouch, have been engineered to promote vascularization and retrieval. While promising, these devices face practical challenges, including limited engraftment capacity and fibrotic encapsulation in human trials (18).

Parallel to physical immune shielding, targeted drug and gene delivery systems have emerged as powerful possibilities for local immunomodulation. These approaches aim to silence pro-inflammatory pathways or enhance beta cell survival without global immunosuppression. Nanoparticles loaded with siRNAs or miRNAs targeting key signaling molecules (e.g., NF-κB, STAT1, CD40) have been used to inhibit T cell activation and preserve graft function in preclinical models. Additionally, polymeric carriers have been developed to deliver anti-inflammatory cytokines, such as IL-10 and TGF-β, to draining lymph nodes or islet grafts. One of the most transformative strategies involves genetic engineering of stem cell–derived beta cells to evade immune detection altogether. Techniques such as CRISPR/Cas9-mediated knockout of HLA class I and II molecules, or overexpression of immune inhibitory ligands like PD-L1 and CD47, have enabled the creation of “hypoimmune” cell lines that resist both adaptive and innate immune attacks (19, 20). These engineered cell lines are currently under evaluation in clinical trials, including the VCTX210 platform, which combines CRISPR-modified stem cell–derived beta cells with macroencapsulation for immunosuppression-free delivery (21).

Pluripotent stem cells either human embryonic (hESCs) or induced pluripotent stem cells (iPSCs), have become a renewable and standardized source of insulin-producing beta cells. Differentiation protocols now replicate pancreatic development stages, producing cells that co-express insulin, C-peptide, and glucose-sensing machinery. Clinical trials by Vertex (VX-880) and ViaCyte (VC-02) have demonstrated partial insulin independence and C-peptide production in patients with T1D (22). Notably, these results have been achieved in some recipients with as little as one dose of stem cell–derived islets, though immunosuppressive regimens are still required. To mitigate immune rejection, future studies are exploring combinations of gene-edited cells, localized drug delivery, and vascularized biomaterial scaffolds. If successful, these strategies could offer scalable, off-the-shelf beta cell replacement therapies that eliminate the need for donor organs and reduce the toxicity of immunosuppression.

In parallel, ML and artificial intelligence (AI) are increasingly integrated into T1D therapy. Most prominently, ML-based closed-loop insulin delivery systems have revolutionized glycemic control by continuously adapting insulin dosing based on CGM data, user input, and predictive algorithms. Automated insulin delivery systems such as Tandem Control-IQ, Medtronic 780G, and CamAPS FX, have demonstrated improvements in HbA1c, time-in-range, and patient-reported outcomes (23). Beyond glycemic management, AI is now being applied to optimize the design of biomaterials and gene circuits, enabling predictive modeling of hydrogel degradation, immune activation, and insulin secretion dynamics (24). Simultaneously, nanotechnology platforms are being developed to deliver drugs, monitor inflammation, and interface directly with islet tissues. Examples include quantum dots for islet imaging, cerium oxide nanoparticles for reactive oxygen species scavenging, and transdermal nanoneedle patches for glucose-triggered insulin release (25, 26). While these tools remain largely in the experimental phase, their integration with biological therapies has the potential to transform precision medicine in T1D.

Although diverse in mechanism and application, all current therapeutic strategies converge on a central challenge: the need to overcome immune rejection. Whether through localized drug release, gene-edited cells, or immunomodulatory biomaterials, achieving immune evasion remains the critical barrier to long-term success in beta cell replacement therapy. A unifying theme across recent innovations is the shift toward immune-protected or immune-tolerant platforms capable of withstanding host immune attack while maintaining physiological insulin secretion. As the field advances, combining technologies such as cell engineering, biomaterials, and ML will be essential to achieve durable, safe, and scalable cures for T1D.

## 2 Immune evasion strategies

To successfully restore beta cell function without lifelong immunosuppression, researchers have been developing immune evasion strategies that enable transplanted cells to evade immune detection while maintaining physiological insulin secretion. These approaches focus on: (i) modifying beta cells to resist immune attack, (ii) designing biomaterial-based encapsulation systems to protect transplanted beta cells, and (iii) leveraging computational models to predict and optimize beta cell replacement therapies. By integrating these advancements, scientists aim to create a more sustainable and durable solution for beta cell replacement therapies in T1D.

### 2.1 Modifying beta cells to resist immune attack

Genetic engineering has emerged as a powerful tool to create immune-evasive beta cells that can escape immune attack while maintaining normal insulin secretion. By leveraging gene-editing technologies such as CRISPR/Cas9, researchers aim to modify beta cells at the molecular level, making them resistant to immune recognition and attack. These strategies primarily focus on removing immune targets, modulating immune signaling, and enhancing cell survival in the hostile autoimmune environment of T1D (**Table 1**).

**Table 1:** Comparison of genetic engineering strategies for making beta cells immune-evasive.

Engineering Strategy	Mechanism & Immune Effects	Advantages	Challenges/Risks
Full HLA class I/II Knockout	Delete genes for all classical HLA class I (e.g. HLA-A, -B, -C via β2M or heavy chain KO) and class II (CIITA or HLA-DQ/DP/DR) on beta cells. Without HLA, CD8^+^ T cells cannot recognize or kill beta cells, and CD4^+^ T cell activation is greatly reduced (20).	Eliminates presentation of autoantigens and alloantigens, preventing T cell-mediated rejection. This can effectively “hide” beta cells from adaptive immune attack.	NK cell activation: Complete absence of HLA class I triggers NK cells (“missing-self” recognition) leading to NK-mediated lysis. Also, loss of all HLA may impair immune interactions needed to fight infections (e.g. inability to present viral antigens). Requires additional modifications to counteract innate immune responses.
Selective HLA Editing (HLA-A/B KO, HLA-C Retained)	Remove the highly immunogenic HLA class I alleles (HLA-A and -B) while retaining HLA-C (and possibly nonpolymorphic class Ib like HLA-E). HLA-C (and HLA-E) provide enough “self” signal to inhibit NK cells, but the most polymorphic antigens (A/B) are absent to minimize T cell recognition (33). Class II is also knocked out to stop CD4 responses.	Avoids NK cell killing by preserving some HLA expression (HLA-C/E can engage NK inhibitory receptors). Reduces likelihood of alloreactive T cells recognizing the graft, since HLA-A and -B mismatches are the main drivers of CD8 responses. In autoimmune context, if dominant epitopes were presented by HLA-A or B, those are eliminated.	Residual T cell detection: Retained HLA-C could still present antigens. Autoreactive or alloreactive T cells restricted to HLA-C may still recognize the beta cells, although HLA-C is less polymorphic. This strategy may not fully prevent immune attack unless combined with other measures (e.g. immunomodulatory ligand expression). It also requires careful selection of which HLA-C alleles to keep (to balance immune evasion vs. residual function).
Overexpression of Immune-Modulatory Proteins	Add genes that actively deliver inhibitory signals to the immune system: HLA-E or HLA-G are non-classical HLA class Ib molecules that interact with inhibitory receptors on NK cells (and some T cells). HLA-E binds NKG2A on NK cells, telling them not to kill (36). HLA-G has immunosuppressive effects on NK and certain T cell subsets. PD-L1 is a ligand for PD-1; its overexpression on beta cells can “turn off” exhausted or activated T cells by engaging PD-1, reducing their proliferation and cytotoxicity (39). CD47 is a “don’t eat me” signal that binds SIRPα on macrophages and dendritic cells, inhibiting phagocytosis and other innate attacks.	Complements HLA editing by protecting against immune mechanisms that bypass MHC. For instance, even if some T cells engage the beta cell, high PD-L1 can suppress their function. HLA-E/G overexpression protects HLA-deficient cells from NK cell lysis, and CD47 reduces clearance by macrophages. These modulators create a local immunosuppressive shield and can be used in combination.	Partial protection: Each immune-modulatory protein addresses one aspect of immunity, so on their own they may not suffice. For example, PD-L1 mainly affects activated T cells (particularly CD8^+^) but might not stop a strong memory T cell response entirely. HLA-E/G help with NK tolerance but do not prevent T cell recognition of other HLA molecules. Overexpression of these proteins must be finely tuned – too much PD-L1 could cause generalized immunosuppression, and ectopic expression of HLA-G might have unknown effects on immune regulation.
Multi-Layered “Hypoimmune” Design (Combine HLA knockout with multiple transgenes)	A comprehensive approach that integrates several modifications to cover all bases. For example, one design removed all HLA class I and II (β2M and CIITA knockout) *and* inserted PD-L1, HLA-G, and CD47 into the cells (39). Another approach kept HLA-C but knocked out HLA-A/B and class II, while adding CD47 and HLA-E (20). The goal is to prevent T cell recognition and attack (via HLA removal and PD-L1), avoid NK cell killing (via HLA-E or HLA-G expression), and prevent phagocytic clearance (via CD47).	Broadly protects against both adaptive and innate immune responses. In preclinical models, such triple/quadruple gene-edited cells (“universal” cells) survived long-term without immunosuppression: e.g. human stem cells edited to remove HLA-A/B/C and CIITA, plus PD-L1, HLA-G, CD47, showed dramatically reduced T and NK cell activation *in vitro* and evaded rejection *in vivo*. This strategy is the most likely to enable transplantation of beta cells without immunosuppressive drugs.	Complexity and safety: Each added modification is another variable, higher chance of off-target effects or altered cell function. Completely “invisible” cells could form tumors or get infected by viruses and go undetected by the immune system. Indeed, even multi-layered HIP cells have shown *residual* low-level immune activation *in vitro*, meaning immune evasion might not be absolute. There are also manufacturing challenges in making multiple precise edits and ensuring stability of edited cells. Regulatory agencies will closely scrutinize such cells given the extent of manipulation.

**Figure 3:**
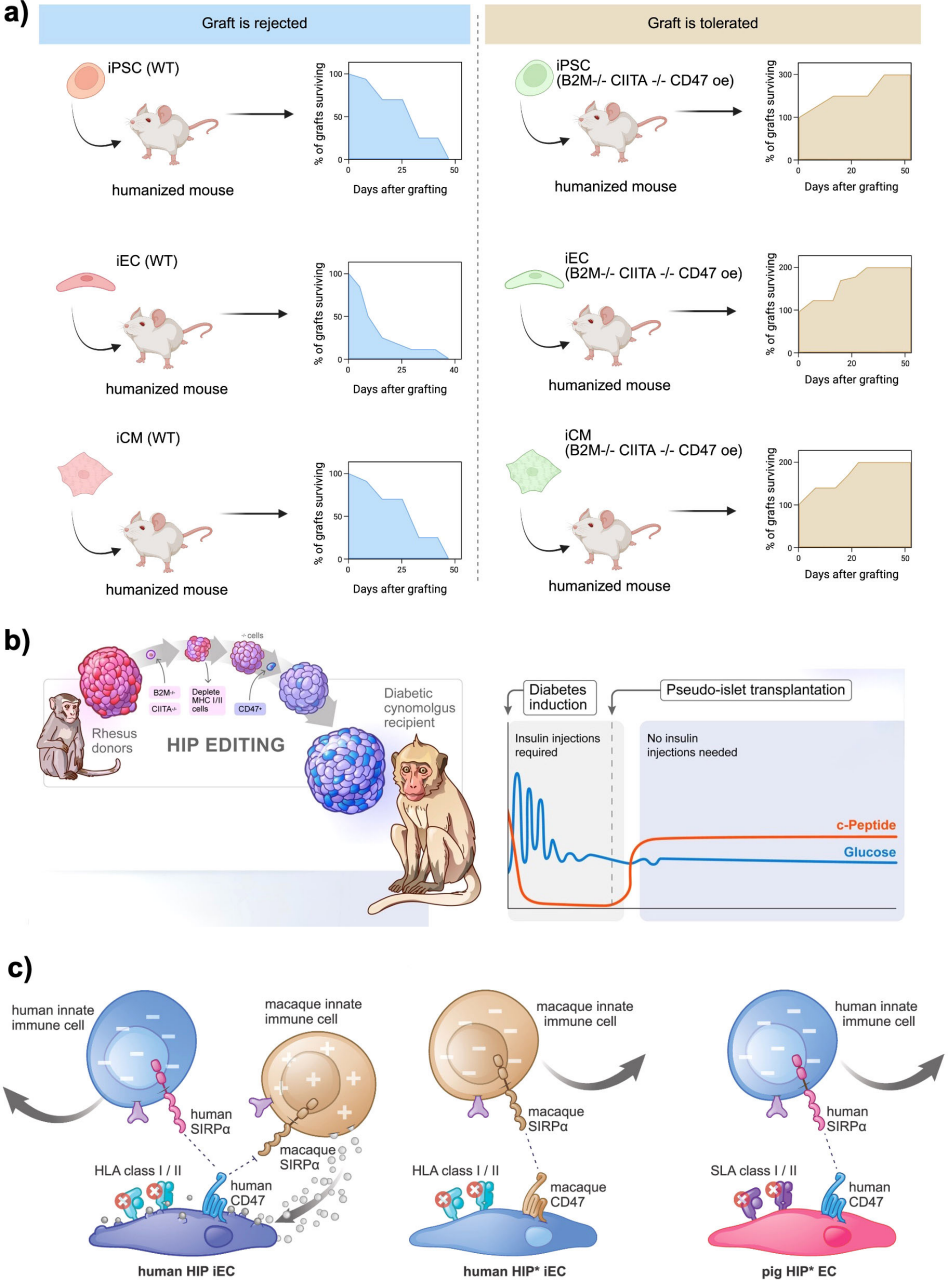
Genetic engineering approaches to generate immune−evasive beta cell grafts. **(a)** *In vivo* survival of wild−type (WT) versus hypoimmunogenic (“B2M^-^/^-^ CIITA^-^/^-^ CD47 OE) human iPSC−, endothelial− (iEC) and cardiomyocyte−like (iCM) derivatives transplanted into humanized mice. WT grafts are uniformly rejected within 4–6 weeks, whereas cells edited to knock out B2M and CIITA and overexpress CD47 show progressive graft expansion and durable survival over 50 days. Created in https://BioRender.com **(b)** HIP editing of rhesus macaque islets for allogeneic transplantation into diabetic cynomolgus monkeys. Primary islets from MHC−matched donors are sequentially modified B2M^-^/^-^, CIITA^-^/^-^, CD47 overexpression and then transplanted under the omental pouch. Recipients become insulin independent, with sustained C−peptide production and stable glycemia post−transplant. Reprinted from (34) **(c)** Inhibition of polymorphonuclear cell (PMN)–mediated xenotoxicity against hypoimmune endothelial cells (HIP* ECs). Human HIP* ECs (HLA-deficient, CD47-overexpressing) are rapidly killed by rhesus PMNs *in vitro* and *in vivo*, even in blood type–matched non-human primates but survive when PMN function is blocked by a multi-drug regimen or when engineered to overexpress PMN-inhibitory ligands CD99 and CD200, enabling prolonged xenograft survival. Reprinted from (35).

CRISPR/Cas9 gene-editing technology uses a guide RNA to target the Cas9 nuclease to specific DNA sequences, inducing a double-strand break. In beta cell engineering, CRISPR is used to knock out genes (i.e., deleting HLA-A/B genes or β2M to remove HLA class I surface expression) or to knock in protective genes (i.e., inserting a PD-L1 or CD47 transgene) (**Figure 3**) (19, 20). For HLA class I, deleting β2-microglobulin (B2M) prevents cell-surface expression of all class I heavy chains (HLA-A, -B, -C) (27). Similarly, disrupting the Class II transactivator (CIITA) abolishes HLA class II expression (28). Leite et al. showed that hPSC-derived beta cells lacking class I provoke significantly weaker CD8^+^ T cell activation *in vitro* than unedited cells (29). Additionally, HLA-I knockout stem cell–derived beta cells elicited lower T cell activation markers (CD25/CD69) when co-cultured with autologous T cells. This confirms that removing HLA class I can prevent recognition by alloreactive T cells. However, complete HLA-I loss creates a new problem of natural killer (NK) cell–mediated rejection. NK cells detect the absence of self MHC I (“missing-self” mechanism) and can lyse HLA-null cells (30). Indeed, B2M-knockout hPSCs became vulnerable to NK cell killing in several studies (20). Furthermore, a graft with no MHC I or MHC II cannot present viral or malignant antigens to the host immune system, raising safety concerns (31, 32). To overcome NK attack while retaining T cell evasion, researchers pursue selective HLA editing rather than total knockout. One strategy is to retain a subset of HLA class I that provides NK-inhibitory signals but minimally triggers T cells. For instance, Xu et al. knocked out HLA-A and HLA-B in iPSCs but left HLA-C intact (33). Engineered cells with only HLA-C could evade CD8^+^ T cells and NK cells *in vitro*. The rationale is that HLA-C is less polymorphic/immunogenic and can engage inhibitory KIR receptors on NK cells, deterring NK attack, while the highly immunogenic HLA-A/B are absent.

Another approach is to add back non-polymorphic HLA molecules to send “self” signals to NK cells. For example, Gornalusse et al. fused B2M to HLA-E and expressed this in B2M-knockout hPSCs (36). HLA-E is a non-classical class I molecule that binds NK inhibitory receptor NKG2A. These B2M–HLA-E–expressing cells were protected from both CD8^+^ T cells and NK cells. Similarly, Shi et al. created a membrane-bound B2M–HLA-G fusion in HLA-I–null hPSCs, achieving hypoimmunogenic cells with reduced NK activation (37). Importantly, HLA-E or HLA-G provision preserves an “NK brake” without restoring the highly antigenic classical HLA molecules. Retaining some HLA expression may also allow the graft to present infection or tumor antigens to the host immune system if needed, a potential safety advantage over completely “invisible” cells. On the other hand, even retained HLA-C or introduced HLA-E/G could present some peptides and permit residual immune recognition (e.g. HLA-C can present viral peptides, and not all NK cells are inhibited by HLA-E/G) (31, 32, 37). Thus, the balance between removing immunogenic HLA and keeping enough “self” signal is crucial.

Notably, most HLA-editing strategies target both class I and II. Stem cell–derived beta cells upregulate Class I upon differentiation and inflammation, and can express Class II under inflammatory conditions, which would present antigens to CD4^+^ T helper cells (38). Therefore, an ideal hypoimmunogenic edit often combines class I knockout (or modification) with class II knockout (via CIITA deletion) to avoid both CD8^+^ and CD4^+^ T cell recognition. In this context, the foundational work by Deuse et al. demonstrated that iPSCs could be engineered to evade immune rejection by knocking out both HLA class I and II genes and overexpressing CD47, a “don’t eat me” signal that inhibits phagocytosis by macrophages (20). These hypoimmune (HIP) iPSCs successfully differentiated into endothelial cells, smooth muscle cells, and cardiomyocytes and survived in fully immunocompetent allogeneic recipients without immunosuppression (**Figure 3a**). Further, Han et al. introduced PD-L1 and HLA-G to actively suppress immune surveillance by T cells and NK cells in addition to HLA-A/B/C and HLA-II knock-out (39). Their approach demonstrated a multi-layered strategy to control both adaptive and innate immunity, paving the way for “off-the-shelf” universal cell products.

Building on these efforts, Hu et al. applied similar genetic modifications to primary human pancreatic islets, creating HIP pseudoislets by knocking out HLA-I and HLA-II while overexpressing CD47 (40). These engineered islets successfully engrafted in immunocompetent, allogeneic, diabetic humanized mice, restoring glucose homeostasis without the need for immunosuppression. The same group extended this research by demonstrating that HIP pseudoislets could achieve insulin independence after allogeneic transplantation into a fully immunocompetent, diabetic non-human primate without requiring immunosuppressive drugs (**Figure 3b**) (34). Further validating this approach, Hu et al. showed that HIP iPSCs, derived from rhesus macaques and genetically modified to lack HLA-I and HLA-II, survived long-term in fully immunocompetent, allogeneic rhesus macaques (41). In a recent study, Hu et al. addressed the previously challenging obstacle of xenotransplantation by developing “HIP” iPSC-derived endothelial cells (**Figure 3c**). These cells were engineered to combine the knockout of B2M and CIITA genes with species-matched CD47 and, most notably, the PMN-inhibitory ligands CD99 and CD200. In cynomolgus monkeys, transient pharmacologic blockade or permanent genetic silencing of polymorphonuclear cell cytotoxicity facilitated the durable engraftment of these HIP ECs. Furthermore, analogous pig HIP* ECs demonstrated resistance to adaptive and innate human immune attacks *in vitro*. By identifying neutrophils as a primary, yet druggable, barrier to cross-species grafting, this study not only enhances the prospects of scalable pig-to-human islet replacement but also proposes a vascular “companion cell” strategy that could stabilize HIP beta-cell grafts in future composite transplant designs (35).

RNA interference (RNAi) offers a versatile means to modulate alloantigen expression and promote immune tolerance in cell therapies. Like CRISPR/Cas9, RNAi can abrogate immunogenic targets by exploiting short double-stranded RNAs to recruit the endogenous RNA-induced silencing complex, thereby degrading specific mRNA transcripts or blocking their translation. This post-transcriptional silencing can eliminate surface MHC molecules without introducing permanent genomic alterations or the attendant risks of gene editing.

In vascularized grafts, host responses to mismatched HLA on endothelial cells (ECs) often precipitate rejection. Ex vivo delivery of CIITA-targeted siRNA to donor vessels abolished HLA-II expression in ECs and prevented their destruction by allogeneic PBMCs in immunodeficient mice (42). For more durable suppression, lentiviral vectors encoding shRNA against B2M achieve sustained HLA-I knockdown. Residual HLA-I levels suffice to inhibit NK-cell–mediated lysis while preventing CD8^+^ T-cell activation (43). Such shRNA approaches have also been applied to iPSCs, yielding HLA-I–reduced megakaryocytes capable of generating functional platelets in murine models of platelet refractoriness (44).

RNAi avoids permanent DNA cleavage and can be titrated or withdrawn, making it well suited for proof-of-concept and temporally defined applications. However, its requirement for repeated administration and variable knockdown efficiency may limit durability. In contrast, CRISPR/Cas9 offers heritable edits but carries increased risk of off-target genomic alterations. Together, RNAi and CRISPR/Cas9 form a complementary toolkit for finely tuning β-cell immunogenicity.

Understanding the genetic mechanisms underlying immune-mediated beta cell destruction is crucial for developing effective cell replacement therapies for T1D. While previous studies have focused on targeted gene modifications aimed at reducing immune recognition, recent advances in genome-wide CRISPR screening have enabled the unbiased identification of novel genetic factors that regulate beta cell susceptibility to immune attack. Two key studies, one conducted in a mouse model of T1D and the other in human stem cell-derived islets (SC-islets), have provided valuable insights into the molecular determinants of immune evasion and potential strategies for engineering immune-protected beta cells.

**Figure 4:**
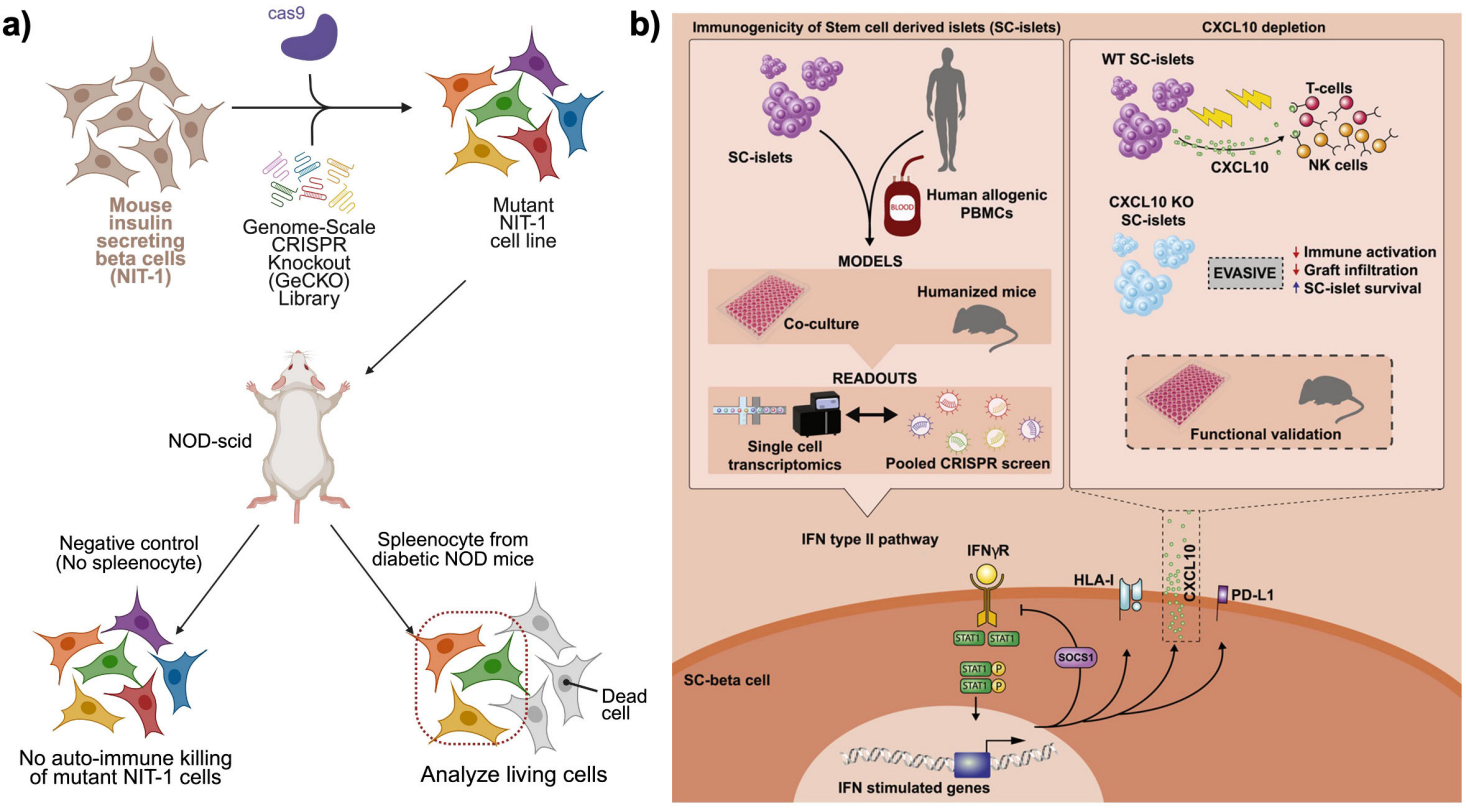
CRISPR knock-out library approaches to find candidate gene hits for immune−evasive beta cell grafts. **(a)** Pooled genome−scale CRISPR knockout (GeCKO) screen in mouse NIT−1 insulinoma cells to identify genes mediating autoimmune killing. A library of mutant NIT−1 cells is transplanted into NOD−scid mice, followed by transfer of diabetic NOD splenocytes. Surviving beta cells are recovered and sequenced to reveal candidate gene disruptions that confer resistance to autoreactive T and NK cell attack. Created in https://BioRender.com **(b)** Dissection of the CXCL10−mediated IFNγ response in human stem cell–derived islets (SC−islets). Combined readouts including co−culture with PBMCs, humanized mouse engraftment, single−cell transcriptomics, and pooled CRISPR screens, identify IFNγ−stimulated CXCL10 as a key chemokine driving T cell and NK cell infiltration. CXCL10 knockout SC−islets exhibit reduced immune activation, diminished graft infiltration, and enhanced SC−islet survival in functional validation assays. Reprinted from (45).

Cai et al. employed a genome-wide CRISPR knockout (GeCKO) screen to identify genetic modifications that render beta cells resistant to autoimmune destruction in a mouse model of T1D (**Figure 4a**) (46). Using the NIT-1 beta cell line, derived from non-obese diabetic (NOD) mice, GeCKO library was introduced to generate a diverse pool of gene-deleted beta cells. These mutant cells were transplanted into immunodeficient NOD-scid mice, which subsequently received splenocytes from diabetic NOD mice to simulate an autoimmune attack. After eight weeks, the surviving beta cells were isolated, and sequencing of the retained CRISPR guide RNAs revealed a small set of genes whose knockout conferred protection against immune-mediated destruction. Among these, Rnls (Renalase) emerged as a particularly strong candidate. Deletion of Rnls significantly enhanced beta cell survival in diabetic NOD mice, reducing their vulnerability to cytotoxic CD8^+^ T cell responses. Further studies revealed that Rnls deficiency conferred resistance to ER stress, a key factor in beta cell apoptosis and immune activation. Notably, the study also identified the FDA-approved drug pargyline as an inhibitor of Rnls, demonstrating that pharmacological blockade of Rnls could protect transplanted beta cells and delay diabetes onset in multiple mouse models. This study established Rnls as a novel regulator of beta cell immune resistance and suggested that targeting beta cell stress pathways could be an effective strategy for preventing autoimmune destruction.

Building on these findings, Sintov et al. extended the CRISPR screening approach to human SC-islets in the context of allogeneic transplantation (**Figure 4b**) (45). Unlike the autoimmune model in mice, this study focused on allo-rejection, mimicking the immune response encountered in beta cell transplantation. Human embryonic SC-islets were transduced with a whole-genome CRISPR KO library and subsequently transplanted into NSG-MHC-null humanized mice. To model an allogeneic immune response, human peripheral blood mononuclear cells (PBMCs) from unmatched donors were injected into the mice, initiating an immune attack on the SC-islet grafts. After ten weeks, the surviving SC-islets were retrieved, and sequencing of the enriched and depleted guide RNAs identified key genes that regulated immune rejection. The screen revealed that interferon-gamma (IFNγ)-mediated immune responses played a dominant role in SC-islet rejection, with gene knockouts in this pathway significantly enhancing graft survival. Among the most protective knockouts were CXCL10, TAP1/2, STAT1, and JAK1/2. The deletion of CXCL10, an IFNγ-induced chemokine responsible for recruiting immune cells to the islet graft, substantially reduced immune infiltration and prolonged SC-islet survival in humanized mice. Similarly, knockouts of TAP1/2, which encode key components of the antigen-processing machinery, reduced HLA class I presentation, thereby diminishing immune detection by cytotoxic T cells. Depleting STAT1 and JAK1/2, the central mediators of IFNγ signaling, also proved effective in mitigating immune rejection, further highlighting the critical role of this pathway in transplant immunogenicity.

These genome-wide CRISPR screens provide a comprehensive genetic roadmap for enhancing beta cell survival in both autoimmune diabetes and transplantation settings. The findings suggest two complementary approaches: (i) for autoimmune diabetes, targeting genes such as Rnls to increase beta cell resilience against immune-mediated destruction; and (ii) for allo-transplantation, knocking out genes such as CXCL10 and STAT1 to reduce immune recognition while preserving essential immune functions. By leveraging unbiased genetic screening, these studies offer novel targets for the development of immune-evasive beta cells, paving the way for more durable and clinically viable cell replacement therapies.

### 2.2 Designing biomaterial-based encapsulation systems to protect transplanted beta cells

#### 2.2.1 Encapsulation approaches at different scales

**Figure 5:**
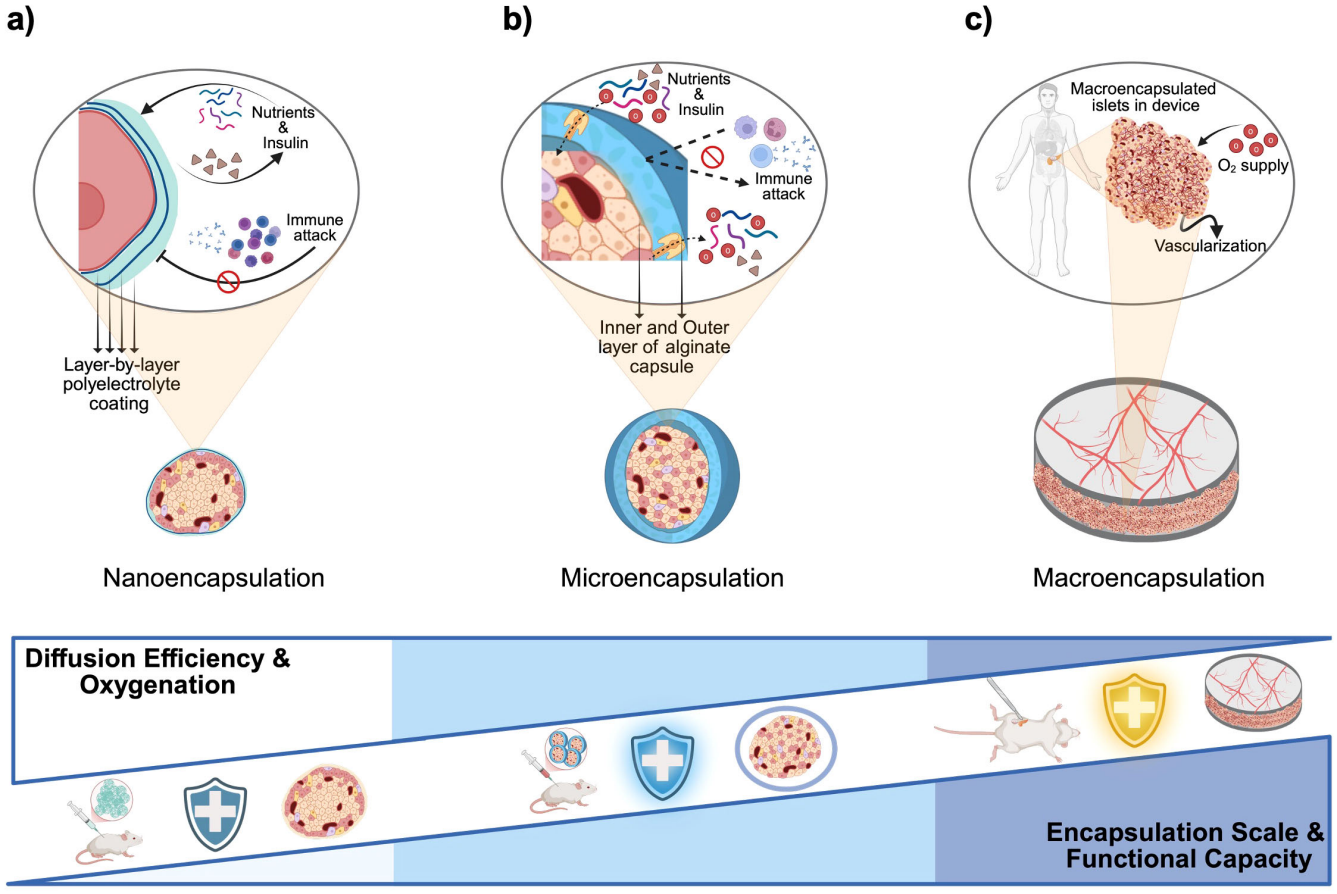
Material-based encapsulations and immunomodulatory strategies for beta cell replacement therapies. **(a)** Nanoencapsulation: Individual islets or pseudoislets are coated with ultrathin polyelectrolyte multilayers (layer-by-layer deposition of alternating charged polymers), forming a nanometer-thick semipermeable barrier that blocks immune cell and antibody access while permitting rapid diffusion of nutrients and insulin. **(b)** Microencapsulation: Small (~200–1,000 µm) hydrogel capsules, typically alginate with an inner and outer layer separated by poly-L-ornithine or poly-L-lysine, enclose individual islets or small clusters; the thicker shell provides robust immunoprotection at the cost of slightly reduced diffusivity. **(c)** Macroencapsulation: Larger, retrievable devices house thousands of islets within one or more chambers; devices rely on host vascularization and may include supplemental oxygen ports or integrated oxygen-generating materials to overcome diffusion limits at this scale. Created in https://BioRender.com.

Encapsulating beta cells in biomaterial devices offers a promising strategy to avoid systemic immunosuppression by isolating the cells from the host immune system while allowing nutrients and insulin to diffuse (47). Early studies showed that semipermeable capsules can protect transplanted tissue from immune attack, significantly prolonging graft survival (48–52). Over the past decades, researchers have developed nano-, micro-, and macro-scale encapsulation strategies to create a system that can replace function of damaged pancreas (**Figure 5**).

Nano-encapsulation involves coating individual cells or islets with an ultra-thin biocompatible layer. A common method is layer-by-layer (LbL) assembly, depositing alternating polyelectrolyte layers (e.g., alginate and poly-L-lysine) to form nanometer-scale membranes (**Figure 5a**) (53). Such conformal coatings tightly wrap the islet like a “second skin” without significantly increasing its size. The protective membrane is typically only a few nanometers thick, favoring bi-directional diffusion of oxygen, nutrients, and insulin (54). This means that nano-encapsulated islets can closely mimic native islet function with minimal diffusion delays. However, nano-encapsulation must achieve complete coverage of the islet surface to be effective. Incomplete or uneven coatings may allow immune cell recognition or entry at exposed spots. Another consideration is that small harmful molecules (pro-inflammatory cytokines, reactive oxygen species) can still permeate thin coatings. To address this, nano-coatings are often combined with biochemical strategies (e.g., anti-inflammatory agents on the coating) (55). Despite these challenges, recent advances have shown promise. For example, PEG-based nanocapsules for porcine islets were transplanted under the kidney capsule of diabetic rats with no significant fibrosis at two weeks and viable islet tissue persisting beyond 100 days (56). Surface chemistry is critical in such systems; adding hydrophilic, “stealth” polymers like PEG on nano-coatings helps reduce protein adsorption and immune activation (57). Researchers have also been exploring optimal nanoparticle shapes and sizes for encapsulation. Zhao et al. reported that rod-shaped nanoparticles show different biodistribution and clearance profiles than spherical ones, affecting how long encapsulated islets remain in the body (58). Overall, nano-encapsulation offers the most compact form of immunoisolation, but ensuring durability and complete immunoprotection at this scale is an ongoing area of development.

Microcapsules are typically 300–800 µm in diameter gel beads (**Figure 5b**), first described by Chang et al., that contain one or a few islets each (59). A classic example is alginate hydrogel microcapsules introduced by Lim and Sun in 1980, which demonstrated that encapsulated islets could survive significantly longer in diabetic rats compared to non-encapsulated controls (60). In a typical microencapsulation process, isolated islets are suspended in liquid alginate and dropped into a calcium chloride solution to form Ca-alginate gel spheres. Often a poly-L-lysine (PLL) coating is added to create a semi-permeable membrane, then an outer alginate layer for biocompatibility. The result is a roughly spherical capsule wherein the islet is immobilized at the center, surrounded by a polymer matrix containing pores. These pores are tuned to allow the free diffusion of glucose, insulin, oxygen, and other small molecules, while blocking direct contact by immune cells and preventing large immune molecules (like antibodies, >150 kDa) from entering. As a result, the hydrogel capsule acts as a tiny immunoisolation chamber for each islet (60).

Microencapsulation has several practical advantages. Because each islet is in its own capsule, the surface area to volume ratio is high, facilitating good nutrient and oxygen exchange for the encapsulated cells (47, 61). The small size also allows delivery via minimally invasive methods (e.g. injection into the peritoneal cavity). In fact, early trials in diabetic primates and patients used intraperitoneal injection of alginate microcapsules. Notably, a study in diabetic cynomolgus monkeys showed that transplanting alginate-PLL microencapsulated porcine islets achieved normal fasting blood glucose and insulin independence for 120 to 804 days in most subjects (62), which was an encouraging proof-of-concept for microcapsules *in vivo.*

Recently, Jing et al. developed an artificial pancreas by engineering PD-L1–expressing β-cells into microspheres (PD-L1 β-MCSs), encapsulating them in an alginate hydrogel, and integrating oxygen-producing Chlorella (PD-L1 β-MCSs-aPancreas). This composite system (i) suppresses immune attack via exosomal PD-L1 released from the microspheres, (ii) reverses hyperglycemia in T1D mouse models, and (iii) enhances β-cell survival within the implant through Chlorella-mediated oxygen supplementation (63). Additionally, Meirigeng et al. demonstrated that alginate microbeads can protect human islets from xenogeneic rejection in immunocompetent mice without the need for immunosuppression. Nevertheless, the grafts ultimately failed, likely due to a macrophage-mediated foreign body reaction (FBR) (64). However, microencapsulation comes with challenges. Scaling up to human cell doses requires a very large number of capsules (on the order of half a million or more for a human transplant), making it difficult to ensure each capsule is intact and retrievable. Capsule size and composition must balance immunoprotection with transport needs. A thicker or denser capsule offers better immune barrier function but can deprive the islet of oxygen and nutrients. Indeed, a major drawback of standard microcapsules lacking internal vasculature is that it does not support blood vessel ingrowth after transplantation (65), which leads to diffusion limits causing hypoxia in the islet core. For example, encapsulating islets in alginate alone exacerbates low oxygen conditions inside the capsule, which can impair islet function. Newer variants like ultra-thin conformal coatings, sometimes considered nano-encapsulation, are being explored to overcome this by reducing capsule thickness (66). However, FBR appears as another concern in this case. The immune system may recognize the alginate capsule itself as a foreign object, leading to fibrotic tissue deposition on the capsule surface over time. This pericapsular fibrotic overgrowth can form a dense barrier that further hinders nutrient diffusion and ultimately isolates the capsule from the bloodstream (67). Despite these issues, microcapsules remain one of the most studied encapsulation mechanisms due to their relative simplicity and proven ability to immunoisolate islets in various models.

Macro-encapsulation devices are larger implants that house hundreds to thousands of islets, or insulin-producing beta cells, together (**Figure 5c**). They often take the form of flat sheets, fibers, or modular chambers that are retrievable. Nutrients and oxygen diffuse from the host tissue across the membrane, and insulin diffuses out to the bloodstream, but immune cells are prevented from crossing the membrane. Macrodevices can be designed with mechanical strength and ports for cell loading, making them suitable for surgical implantation and retrieval for refilling or analysis. Recently, Skrzypek et al. developed a non-degradable microwell-based macroencapsulation device using poly(ether sulfone)/polyvinylpyrrolidone membranes, which prevents islet aggregation and preserves morphology while maintaining glucose-responsive insulin secretion, offering a promising alternative to intrahepatic islet transplantation (68). In another study, Bose et al. introduced an implantable macrodevice that consists of a silicone housing and a nanoporous polymer membrane with a pore size on the order of 0.8–1 µm. The pores are small enough to block immune cell infiltration, while still permitting small molecule exchange. This device supported transplanted cells in immunocompetent mice for over four months without immunosuppression (69). Notably, a specialized zwitterionic polymer coating on the membrane was required to prevent fibrotic tissue encapsulation and proved essential for long-term function (70).

Macrodevices offer greater control over design parameters than microcapsules. Membrane thickness, pore size, and geometry can be engineered to optimize permeability and immune protection. Additionally, macroscale devices can include features like oxygen delivery systems or chambers that can be periodically refilled with fresh cells. They also facilitate monitoring of important parameters, imaging of the graft, or have sampling ports. An important feature of macrodevices is that the retrieval of the entire graft is simpler. If the therapy needs to be halted or the device replaced, a surgeon can remove the implant whereas thousands of widely scattered microcapsules are hard to collect completely.

The trade-off is that macrodevices inherently accommodate more cells in one basket. If the membrane fails at any point (i.e., tears or excessive pore enlargement), a large fraction of cells could be exposed to the immune system at once. Furthermore, the diffusion distance from the device surface to the innermost cells can be significant. Without a blood supply inside the device, cells in the center may suffer from lack of oxygen, especially in the immediate post-implantation period before any host vessels develop around the device. Indeed, devices thicker than a few hundred microns face cell death at their core unless oxygen delivery is enhanced (71). One analysis estimated that a hollow fiber device 200 µm in radius would need to be 17 meters in length to support ~250,000 islet equivalents (IEQ), due to oxygen diffusion limits (72). This highlights the oxygen transport challenge that macro-scale systems must overcome. Additionally, the larger implanted area of macrodevices can provoke a stronger FBR. Fibroblasts and collagen capsules may encapsulate the device, which can isolate it from the host circulation. This fibrotic encapsulation has been a major failure mode in past macrodevice trials, as it effectively suffocates the graft. Modern macrodevices address this via advanced biomaterials and design, using biomaterials that resist fibrosis, or designing the device to encourage vascularization rather than scar formation.

In summary, each encapsulation scale comes with distinct advantages and limitations (**Table 2**). Nano-coatings are extremely cell-friendly but need robust chemistry; microcapsules are proven and modular but require massive quantities and face oxygen limits; macrodevices offer engineered control and retrievability but demand sophisticated solutions to transport and immune challenges. Often, researchers combine approaches (e.g. conformal coating of cells inside a macrodevice) to leverage the benefits of each scale (74).

**Table 2:** Encapsulation scales: nano vs. micro vs. macro.

Encapsulation Type	Description	Advantages	Challenges
Nano-encapsulation	Ultra-thin conformal coatings (tens to hundreds of nm) applied directly on cells or islets (55, 73) Achieved by layer-by-layer deposition of polymers to form a nanomembrane around each islet (53)	Minimal diffusion barrier, allowing rapid nutrient, oxygen, and insulin transport (55) Preserves islet native architecture and size	Difficult to ensure complete, uniform coverage for full immunoisolation Small cytokines or toxins may penetrate thin coatings (partial immunoprotection) Coating stability can be an issue (risk of coating breakage or degradation)
Micro-encapsulation	Spherical hydrogel microcapsules (~200–1000 µm diameter) encapsulating individual islets or small islet clusters (47) Commonly uses alginate or other polymer hydrogels with semipermeable pores (52)	Established technique with simple fabrication (e.g. dripping islets into alginate) (52) High surface-area-to-volume ratio supports nutrient and O_2_ diffusion Each capsule isolates a small number of cells, preventing one failing capsule from exposing all cells	Requires thousands of capsules per transplant (difficult to retrieve all) Capsule volume and hydrogel add bulk, which may hinder insulin diffusion kinetics Fibrotic overgrowth can occur on many small capsules, impacting mass transfer (70)
Macro-encapsulation	Larger implantable devices housing many islets in a single container (millimeter to centimeter scale) Typically consist of cell-loaded chambers sealed with a permselective membrane	Can pack therapeutic cell doses in one unit for easier implantation and retrieval Membrane properties (pore size, thickness) can be precisely controlled and made robust Designed for surgical implantation at a specific site (e.g. subcutaneous pocket)	Limited oxygen and nutrient delivery to cells at device center due to longer diffusion path (72) Larger foreign surface can trigger fibrotic immune response, isolating the device Insulin release may be slower or altered, risking hypoglycemia if kinetics aren’t optimal

#### 2.2.2 Physical immune barrier

Physical immunoisolation is the foundational principle of encapsulation. The capsule material (hydrogel, membrane, etc.) is engineered with a pore size or mesh such that it permits the diffusion of small molecules but blocks larger immune entities. Nutrients (glucose, oxygen, etc.) and secreted insulin are small enough to traverse the membrane freely, ensuring the encapsulated cells can function and the insulin can reach the bloodstream. However, immune cells (i.e., T cells, B cells, macrophages) are whole cells on the order of 5–15 µm and thus cannot pass through a membrane with nanometer-to-micron scale pores. Likewise, large immune proteins such as antibodies and complementary components are typically too large to diffuse into a properly designed capsule (60). By separating the graft from the host tissue, encapsulation prevents immune cell infiltration and direct cell-cell contact between host and graft. This avoids triggers for rejection like antigen presentation and CTL attack. Early transplantation studies in the 1950s already showed that enclosing tissue in a cell-impermeable barrier could significantly prolong graft survival by blocking direct immune recognition (75–77).

Physical isolation must be achieved without impeding the life-sustaining exchanges for the encapsulated beta cells. Effective encapsulation materials have an ideal pore size, which should be large enough to allow rapid nutrient and hormone diffusion, but small enough to block immune effectors. For example, alginate microcapsules with a properly formed PLL membrane are permeable to glucose (180 Da) and insulin (~5.8 kDa), but impermeable to IgG (~150 kDa) and cells, thereby allowing islet function while blocking immune attack (60). This physical barrier grants what is often termed as “immune privileged microenvironment” for to the encapsulated cells, as if they were in an immunologically protected site.

It is important to note that physical shielding is not protective against all immune threats. While cells and large proteins are excluded, smaller cytotoxic molecules (e.g., cytokines) can diffuse through many encapsulation membranes. Additionally, if a capsule’s integrity is compromised, immune cells can infiltrate and rapidly destroy the graft. Therefore, physical shielding is often complemented by biochemical strategies to handle the molecular aspects of immune attack and to improve biocompatibility of the capsule itself.

#### 2.2.3 Biochemical immunoprotection

Biochemical protection refers to strategies that go beyond size-exclusion, aiming to neutralize or avoid the soluble factors of immune attack and modulate the local immune environment. Encapsulation systems in recent years increasingly incorporate biochemical cues for enhanced immunoprotection.

Encapsulation hydrogels can be functionalized with molecules that counteract inflammatory cytokines. Su et al. chemically immobilized a peptide that blocks the interleukin-1 receptor (IL-1Ra) within a PEG hydrogel encapsulating islets. This local IL-1 inhibitor maintained the viability of the encapsulated cells even when exposed to a cocktail of inflammatory cytokines (IL-1β, TNF, IFN-γ) that would normally be highly toxic (78). By soaking up or inhibiting cytokines at the capsule surface, such functionalized capsules provide a biochemical shield in addition to the physical barrier. Kumar et al. showed that sustained release of interleukin-4 (IL-4) and dexamethasone from injectable silk hydrogel promoted polarization of M2 macrophages and preserved transplanted beta cells physiology (79).

The choice of biomaterial can drastically influence the degree of immune activation. Certain natural polysaccharides such as chitosan have also demonstrated immunomodulatory effects through interactions with innate immune receptors and intracellular signaling pathways, which may be leveraged in future encapsulation designs for tolerogenic or immune-instructive applications (80). Encapsulation materials originally were thought to be inert (81), but most provoke FBR (82). Modern designs use hydrophilic, non-fouling polymers like PEG and zwitterionic hydrogels which resist protein adsorption and cell adhesion. Zwitterionic groups have shown especially low immune activation by reducing nonspecific protein binding and cell adhesion more effectively than PEG. Coating an encapsulation membrane with a zwitterionic polymer can thus prevent the cascade of events (protein adsorption → macrophage adhesion → fibrotic capsule formation) that otherwise undergoes immunoisolation. Liu et al. developed a quaternized triazole-based zwitterionic hydrogel to encapsulate islets. The authors showed that zwitterionic hydrogel exhibited minimal protein fouling and greatly reduced fibrotic overgrowth *in vivo* (83). Islets encapsulated in this material survived significantly longer in diabetic mice compared to traditional alginate capsules (49, 50, 52), highlighting how material chemistry confers biochemical immune protection.

**Figure 6:**
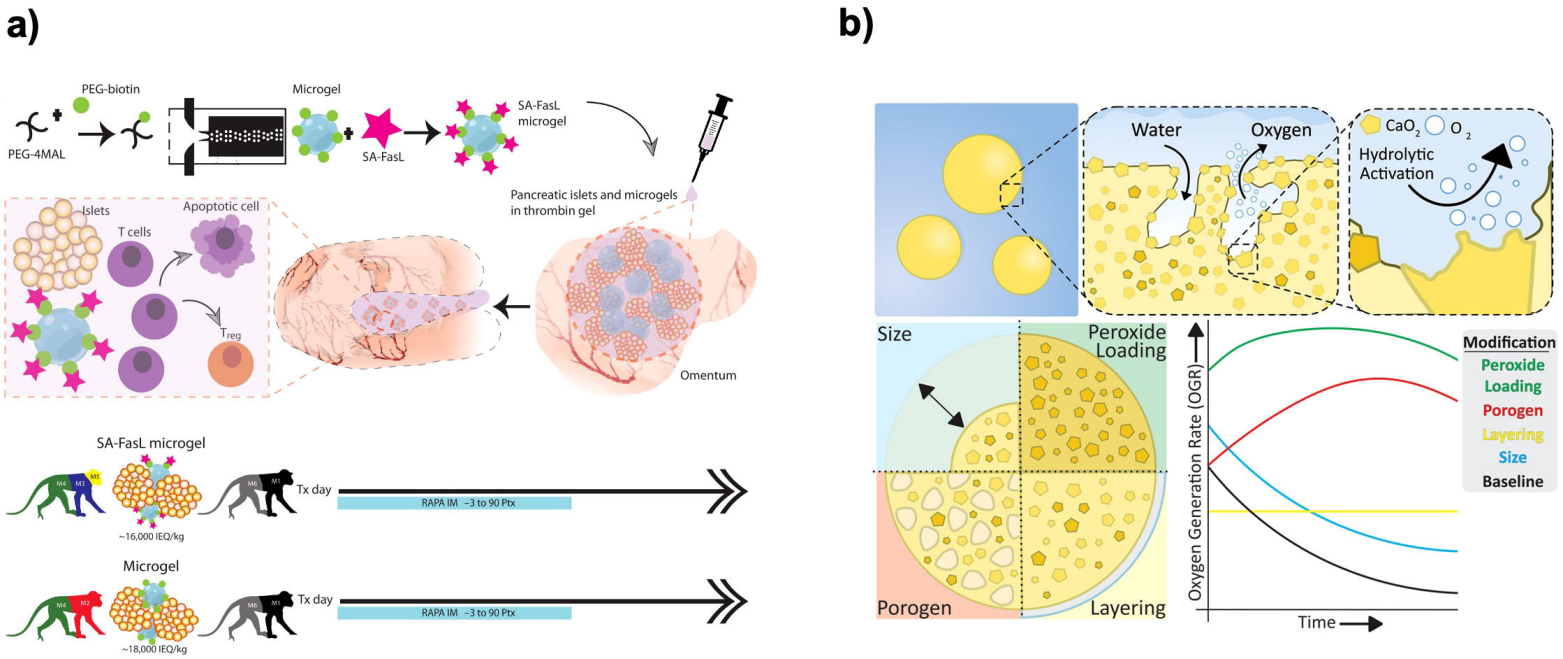
Material-based encapsulations and immunomodulatory strategies for beta cell replacement therapies. **(a)** Local delivery of SA−FasL via synthetic PEG microgels to induce islet allograft tolerance. Fabrication of streptavidin–FasL−presenting microgels and co−immobilization with allogeneic islets in a thrombin gel on the omental pouch. Transient rapamycin regimen (–3 to +90 days) yields robust glycemic control and graft survival in nonhuman primates. Reprinted from (84). **(b)** Modular OxySite oxygen−generating microbeads. Encapsulation of CaO_2_ within PDMS with tunable parameters: peroxide loading, porogen content, bead size, and outer PDMS shell, each modulating *in situ* oxygen−generation kinetics to mitigate hypoxia in avascular implants. Reprinted from (85).

Encapsulation devices can be decorated with molecules that actively engage immune checkpoints to protect the graft. One powerful strategy has been to present the FasL on or with the encapsulated cells. FasL can induce apoptosis of T cells upon binding, effectively killing off the immune cells attempting to attack the graft. Co-transplantation of FasL-expressing cells with islets has been shown to induce long-term graft acceptance without systemic immunosuppression (86). Lei et al. surface-engineered islet surface with a chimeric streptavidin-FasL protein and embedded in a polymer scaffold. They demonstrated that co-transplantation of allogeneic islets with SA-FasL–presenting microgels into the omentum under transient rapamycin treatment induced localized immune tolerance and sustained graft function for over six months in diabetic nonhuman primates, offering a promising immunosuppression-free strategy for beta cell replacement therapy (**Figure 6a**) (84). By inducing apoptosis in infiltrating T cells, the FasL on the graft created a locally immunoprivileged site. Similarly, other immune-modulating ligands like PD-L1 that inhibit T cell activity are being explored to coat encapsulation materials. Coronel et al. functionalized maleimide-terminated four-arm poly(ethylene glycol) (PEG-4MAL) macromers with chimeric streptavidin/programmed cell death-1 (SA-PD-L1) protein to direct “reprogramming” of local immune responses to transplanted pancreatic islets. PEG microgels with SA-PD-L1 on the surface improves local retention of the immunomodulatory agent over three weeks *in vivo*, in combination with a brief rapamycin treatment (17).

Biomimetic delivery platforms have expanded the toolkit for localized co-stimulation blockade. For example, Ma et al. engineered autologous platelets loaded with a triad of immune checkpoint ligands such as PD-L1, Gal-9, and BTLA, which preferentially accumulate in inflamed islet microenvironments and maintain normoglycemia in diabetic mouse models (87). In a complementary approach, Yang et al. fabricated artificial extracellular vesicles (aEVs) displaying PD-L1 and Gal-9 on their surface. These aEVs induce T-cell apoptosis and foster regulatory T-cell differentiation, resulting in enhanced β-cell survival and immune protection in new-onset T1D mice (88). Together, these innovative platforms illustrate how targeted delivery of checkpoint molecules can synergize with encapsulation strategies to create a multifaceted barrier against allo- and autoimmunity.

Beyond encapsulation and pharmacological release systems, genetic engineering of donor cells to express co-stimulation inhibitors offers a potent, localized form of immunomodulation. LEA29Y (belatacept), a high-affinity CTLA-4-Ig analogue, has been driven by the porcine insulin promoter in transgenic pigs to achieve β-cell–specific expression (89). Neonatal islet cell clusters (ICCs) from INS-LEA29Y transgenic pigs normalized blood glucose in streptozotocin-diabetic NOD-scid IL2Rγ^-^/^-^ mice and, following adoptive transfer of human peripheral blood mononuclear cells, remained protected from rejection throughout a 30-day observation period, whereas 80% of wild-type ICC grafts were rejected (90, 91). Local LEA29Y concentrations within the graft microenvironment were 30–100× lower than systemic belatacept regimens, minimizing off-target immunosuppression while maintaining durable T-cell co-stimulation blockade (92). This strategy complements biomaterial-based encapsulation, enabling a multifaceted barrier against allo- and xenogeneic attack without the toxicity associated with systemic immunosuppression.

In summary, biochemical immunoprotection strategies recognize that even if immune cells are physically prevented from reaching their target, the biochemical signals (inflammatory cytokines) of the immune system can still diffuse and exert their effects. By neutralizing those signals or making the graft invisible or tolerogenic to the immune system, these strategies greatly improve the effectiveness of encapsulation. The most cutting-edge encapsulation systems therefore combine physical immunoisolation with biochemical modulation, creating a multi-faceted protective niche for the transplanted beta cells.

### 2.3 Key challenges and recent advances in biomaterials and beta cell replacement therapy

Encapsulated beta cell systems face several critical challenges that must be addressed for long-term success. The major issues include immune rejection and foreign body responses, insufficient oxygenation, poor vascular integration, and various forms of cellular stress on the encapsulated cells. Over the past decade, there have been significant breakthroughs in encapsulation technology. Researchers have addressed many limitations of earlier designs through innovative materials and engineering strategies.

#### 2.3.1 Fibrosis and immune reactions

The host immune system can mount responses against both the graft cells and the encapsulation material. While encapsulation prevents direct immune cell attack, the implant can still trigger FBR. This typically involves macrophages and fibroblasts accumulating on the device and forming a collagenous capsule (fibrosis) around it. A dense fibrotic layer will isolate the encapsulated cells from the bloodstream, depriving them of nutrients and compromising the device function. However, when the encapsulation membrane is not perfectly providing immunoisolation, small antigens can leak out and sensitize the host, leading to antibodies against the graft. Immune rejection in encapsulation thus often manifests as device failure over time where the graft works initially, but immune-mediated fibrosis or subtle leakage-induced reactions progressively choke off its function.

To address this challenge, Vegas et al. performed a large-scale combinatorial study of alginate derivatives, essentially synthesizing a library of over 700 alginate variants with different chemical modifications and screened them for biocompatibility (93). They identified a particular alginate modification with triazole-thiomorpholine dioxide, a zwitterionic moiety, that dramatically reduced fibrosis around capsules. Using this chemistry, they achieved long-term survival of encapsulated islet grafts in immunocompetent mice and even in non-human primates. This work was a landmark showing that materials could be engineered at the molecular level to be invisible to the immune system. Similarly, other groups have developed zwitterionic hydrogels and PEGylated capsules that show minimal cellular overgrowth and extended biocompatibility *in vivo* (83, 94).

The size of capsules can influence immune reaction. Smaller capsules (or thinner devices) seem to cause less FBR, possibly by altering macrophage fusion and foreign body giant cell formation. Veiseh et al. showed that spherical capsules below about 0.5 mm induced fewer macrophage-driven giant cells than larger ones (95). This is linked to the colony stimulating factor-1 (CSF-1) pathway in macrophages (96). Therefore, minimizing capsule size without compromising functionality is advantageous. Likewise, shaping macrodevices to have high surface curvature or porous surfaces can reduce continuous flat areas that encourage fibrosis. For instance, adding 30–40 µm pores on an implant surface was shown to reduce fibrotic tissue and macrophage giant cells, instead promoting more constructive tissue integration (97).

#### 2.3.2 Vascularization, oxygenation, and nutrient supply

Encapsulated beta cells are highly metabolic and require adequate oxygen and nutrients. In native islets, each islet is permeated by a dense vasculature network providing oxygen (98). In encapsulation, this direct perfusion is absent due to a lack of immediate angiogenesis after implantation (65, 99, 100). The cells rely on diffusion through the encapsulating material and any surrounding tissue fluid. Oxygen is the scarcest resource because of its low solubility and the relatively long diffusion distances that can occur. The result is that encapsulated cells often experience hypoxia, which can lead to cell death or impaired insulin secretion since beta cells are very sensitive to low oxygen and reduce insulin output in response to oxygen deprivation (101). In larger devices, cells in the core may necrose, and even in microcapsules, the center of the islet can be hypoxic post-transplant until new capillaries form around the capsule. Insufficient oxygen was identified as a cause of graft failure, as the devices must be only a few hundred microns thick for oxygen to adequately reach interior cells (102). This severely limits how many cells can be packed within a device to ensure sufficient oxygen delivery.

One of the more successful strategies has been to pre-vascularize the implant site. Techniques include creating a subcutaneous pouch or space and inducing angiogenesis there before introducing the encapsulated cells. For example, inserting a dummy catheter or scaffold subcutaneously for a few weeks prompts the body to form new blood vessels in response to the foreign presence (103), and removing it leaves a highly vascularized tissue pocket. When encapsulated islets are then implanted into this preconditioned site, they immediately benefit from the proximity of blood supply and can establish function without a prolonged ischemic period. Additionally, advancements in 3D printing have allowed creation of precise architectures that can improve mass transfer and provide channels for blood vessel ingrowth. Farina et al. developed a novel 3D printed and functionalized polylactic acid (PLA) encapsulation system for subcutaneous engraftment of beta cells (104). As a result, the device protected the encapsulated islets from acute hypoxia and kept them functional *in vivo*.

Another approach for oxygenation of the implant is *in situ* oxygen generation. Calcium peroxide (CaO_2_) is a compound that, upon contact with water, releases oxygen and calcium hydroxide (CaOH)_2_. By embedding CaO_2_ particles in a hydrophobic matrix to control their rate of reaction, a slow oxygen release system can be produced (**Figure 6b**) (85). Pedraza et al. reported that a PDMS disk loaded with CaO_2_ was attached to an islet encapsulation device and provided supplemental oxygen for over 40 days *in vivo* (100). This significantly improved graft survival in the critical early post-implant period. Additionally, encapsulation matrices can be loaded with oxygen-rich liquids. Perfluorocarbons (PFCs) dissolve large amounts of oxygen and mixing a PFC emulsion into an alginate capsule can roughly double the oxygen solubility within the capsule. While PFC eventually diffuses out or gets metabolized, it can provide a buffer against hypoxic dips (105).

#### 2.3.3 Stress responses

Encapsulated beta cells are subjected to various stresses that compromise their survival and functionality. One such stress is the absence of their native extracellular matrix (ECM) and cell interactions. Beta cells might not receive the anchorage signals they normally get from basement membrane components within a capsule. Beta cells are accustomed to an environment rich in collagen IV, laminin, and fibronectin that engage integrin receptors on their surface and promote cell survival and insulin secretion (106). Encapsulation in a plain alginate gel, which is largely inert and lacks these ligands, can lead to beta cells feeling “homeless,” triggering anoikis, cell death due to loss of attachment, or at least a reduction in function. To avoid this, one straightforward solution is to incorporate ECM molecules or analogs in the encapsulation matrix. Studies have shown that including ECM components can improve outcomes such as adding collagen or RGD peptides to capsules improved islet viability and insulin release in culture (107, 108). Many recent hydrogel designs are hybrid, combining biomaterials with ECM proteins like collagen, laminin or with short peptides that mimic their cell-binding domains (e.g., RGD from fibronectin, IKVAV from laminin). Bal et al. decorated PEG hydrogels with RGD and IKVAV peptides to improve beta cell function (109), and showed that ECM-derived peptides improved beta cells function in response to altered glucose levels in the physiological environment. Additionally, hyaluronic acid, a natural ECM glycosaminoglycan, has also been mixed with alginate to provide a more cell-friendly microenvironment. The encapsulated islets “feel” more at home and are less prone to anoikis or dysfunction. Some groups encapsulate islets within a sandwich of an inner layer containing ECM proteins and an outer pure alginate layer for immunity, thus separating the roles of cell support and immunoisolation (110–112).

Another stress is mechanical stress or confinement. Some encapsulation methods like tight conformal coatings might exert slight compression on islets, or gels might contract and squeeze cells. Beta cells can be sensitive to mechanical forces where too much compression can impair insulin granule exocytosis or cause cell damage. To avoid harmful compression, hydrogels can be formulated to have appropriate stiffness. A very stiff capsule might not allow islet expansion (islets can swell slightly) and can transmit external forces. A too-soft capsule might compromise integrity. Karaoglu et al. optimized mechanical properties of GelMA hydrogels through artificial neural network (113). By using the trained network, crosslinking density can be fine-tuned to achieve a modulus similar to soft tissue that is ideal, thus the beta cells are not overly constrained. Additionally, making capsules slightly larger than the islet (for microcapsules) prevents any significant compressive pressure on the islet surface. Novel “shape-fitting” conformal coatings must carefully control deposition to avoid shrinking onto the islet.

Encapsulated cells can also face metabolic stress. If mass transfer is suboptimal, waste products (like CO_2_ and lactic acid) might accumulate locally, or glucose might take longer to diffuse, causing transient local highs or lows. High glucose exposure without proper pulsatility could exhaust beta cells or induce ER stress as they overwork (114). Similarly, being in a foreign environment might alter the paracrine signaling within an islet, where alpha, beta, and delta cells signal to each other. Encapsulation does not inherently disrupt islet architecture. However, if single beta cells are encapsulated in extreme nano-encapsulation cases, they lose those intra-islet signals (115). To reduce metabolic stress and delays, researchers sometimes create multi-porous capsules that have two tiers of porosity. While large pores are for fast glucose/insulin diffusion and small pores are for immunity. This can blunt extreme glucose concentration gradients. Also, placing encapsulated cells near richly perfused areas ensures rapid removal of waste and distribution of insulin, so the cells are not immersed in their secretions or waste products (116).

Finally, inflammatory stress from the host can still affect encapsulated cells. Even if immune cells do not contact them, diffusible factors like cytokines (IFN-γ, IL-1β, TNF-α) can penetrate and induce stress pathways in beta cells. These cytokines provoke oxidative stress and apoptosis in beta cells, contributing to graft failure if not mitigated (117, 118). To combat inflammatory and oxidative stress, encapsulation matrices have been infused with cytoprotective agents. For example, a capsule might include slow-releasing antioxidants (like catalase, N-acetylcysteine or small molecules) to neutralize reactive oxygen species that diffuse in due to inflammatory cytokines. Reys et al. investigated the effect of fucoidan, an antioxidant derived from *Fucus vesiculosus* (FF), on encapsulated beta cell survival and function (119). They showed that both viability and glucose responsiveness of beta cells in fucoidan incorporated alginate microcapsules are significantly higher compared to beta cells encapsulated in alginate alone.

Each of these challenges is interrelated, and solutions often address multiple challenges at once. For example, improving vascularization helps with oxygen and relieves some immune pressure by promoting healthy tissue integration. The field now recognizes that a comprehensive design addressing all these factors is needed. As we design better encapsulation systems, we are effectively learning to bioengineer a microenvironment that replicates key features of the pancreas in a protected fashion. The final piece of this puzzle leverages modern computational tools, which are discussed in the next section, to handle the complexity of optimizing these multifactorial designs.

## 3 Leveraging computational models to predict and optimize beta cell replacement therapies

Given the complexity and interplay between immune evasion and biomaterial encapsulation strategies, computational modeling and ML approaches provide essential tools for optimizing therapeutic outcomes and overcoming existing barriers in beta cell replacement therapy.

Mathematical modeling of glucose-insulin dynamics has been pivotal in advancing predictive control algorithms underpinning closed-loop insulin delivery systems, widely known as artificial pancreas systems. These computationally efficient models aim to maintain blood glucose concentrations within a safe glycemic range in individuals with T1D, predicting glucose concentration trajectories in response to insulin administration, dietary carbohydrate intake, and physical activity. Recent developments include digital twins, virtual replicas of individuals with T1D, constructed using measurable physiological parameters, enabling simulation of diverse clinical scenarios and guiding treatment decisions.

Mathematical models have significantly advanced preclinical research by enabling in silico trials. A noteworthy milestone was the acceptance by the FDA of the University of Virginia/Padova metabolic simulator as a surrogate for animal studies, underscoring the robustness and predictive accuracy of these models (120–122). Driven by the need to assess multivariable artificial pancreas systems capable of managing glucose excursions induced by exercise, the diabetes mathematical models have been expanded to incorporate physical activity effects (123, 124). Furthermore, the simulations software has been further enhanced by integrating additional physiological signals as outputs, thus supporting in silico testing of fully automated, multivariable closed-loop insulin delivery systems that effectively manage unannounced meals and physical activities.

In parallel, mathematical models have also been extensively developed to understand, diagnose, and predict disease trajectories in Type 2 Diabetes (T2D). A significant advancement has been the creation of minimal models capable of estimating insulin sensitivity and glucose effectiveness based on glucose tolerance or mixed-meal tolerance tests (125–128). Their structural simplicity and parameter estimation capabilities have driven widespread adoption (129). Extensions of these minimal models include two-compartment systems accounting for rapid intravenous glucose dynamics, bidirectional glucose-insulin feedback mechanisms, and sophisticated parametric descriptions of glucose absorption rates following meals (130–132). Additionally, complex physiological models addressing prehepatic insulin secretion, incretin hormone effects on insulin and glucagon secretion, and as glucagon and C-peptide kinetics have been proposed for a deeper physiological insight into T2D (133–135). Further, dynamic modeling of beta cell mass provides quantitative descriptions of T2D progression, complemented by systems pharmacology approaches aimed at elucidating pharmacokinetic and pharmacodynamic characteristics of antidiabetic medications, ultimately aiding in therapeutic decision-making (136, 137).

For T1D specifically, mathematical models have also explored critical pathophysiological aspects involved in disease onset and progression (138–142). The Copenhagen model, for example, suggests defective clearance of apoptotic beta cells, leading to necrosis, as a potential trigger of autoimmune processes in non-obese diabetic mice (143). This model hinges on a concept of bidirectional stability between healthy and diseased states, with transition mechanisms driven by macrophage clearance dynamics (144). Other models have specifically investigated the role of T_regs_ cells in autoimmune responses, with detailed mathematical descriptions of activated, memory, and effector T cell populations during late-stage autoimmune progression (145, 146). Such models help elucidate why memory T cells exhibit reduced avidity and provide weaker protection for beta cells over time (147–149). Additionally, models have examined the impact of ER stress on beta cell apoptosis and the role of viral infections in autoimmune diabetes progression in murine models (150–160). These sophisticated approaches primarily employ nonlinear ordinary differential equations to represent immune cell dynamics. Recently, advanced agent-based spatiotemporal models have been proposed, utilizing data from human pancreatic samples collected near the onset of T1D to study insulitis, the characteristic inflammation in pancreatic islets during disease initiation (161, 162).

Emerging techniques in molecular profiling, high-throughput sequencing, and advances in ML, supported by high-performance computational platforms, have further expanded modeling approaches to dissect T1D pathogenesis at a molecular and cellular level. ML combined with genomic profiling enables stratification of early-stage T1D subjects into subgroups exhibiting strong or weak immunotherapeutic responses, facilitating predictions of clinical outcomes and guiding personalized interventions (163). Advanced ML algorithms, such as extreme gradient boosting, a type of boosted decision-tree model, have been employed on islet gene-expression data obtained via single-cell RNA sequencing. These models provide unprecedented resolution into disease progression at a single-cell granularity, distinguishing autoantibody-positive individuals from healthy controls (164).

Complementary advances in computer vision allow identification and differentiation of alpha and beta cells within live, intact human islets without the need for immunostaining. This non-invasive imaging technology provides valuable insights into dynamic cellular events associated with T1D progression (165). ML has also demonstrated potential for identifying biomarkers predictive of islet allograft immune rejection or tolerance, significantly informing transplantation strategies. Furthermore, these techniques facilitate comprehensive immunological profiling, revealing novel pathogenic mechanisms and enabling refined, pathogenesis-based patient stratification (163–166). Collectively, these breakthroughs represent significant strides toward precision medicine in T1D management (167).

AI and ML techniques further contribute to understanding the epidemiology, progression, and clinical presentation of T1D, surpassing conventional statistical methods in handling the heterogeneity inherent in extensive patient datasets. These methods underpin the development of sophisticated digital twins for predictive in silico trials across various physiological conditions, aiding in novel drug target discovery and biomarker identification (163). Future applications of AI and ML may accelerate identification of existing immunotherapeutic agents suitable for repurposing for ameliorating T1D and facilitate novel drug discovery by integrating genomic, transcriptomic, chemical, and drug-target interaction data. Methods capable of predicting therapeutic non-responsiveness are especially valuable in advancing personalized therapeutic strategies. Next-generation digital twins that incorporate genomic, transcriptomic, and metabolomic datasets hold significant potential for personalized therapeutic simulations and in silico evaluation of immunotherapies.

Despite these advancements, several challenges must be addressed to harness the transformative potential of AI and ML for T1D treatment and cure. Algorithms need robustness against heterogeneous and incomplete real-world data and require interpretability and transparency to instill confidence in clinical decision-making. Ensuring the generalizability of these models across diverse subpopulations remains critical. As AI and ML increasingly influence clinical practice, adherence to standards that promote accessibility, reproducibility, and transparency will be essential, ensuring that these advances are effectively translated to clinic and enhance the wellbeing of individuals with T1D.

## 4 Clinical trials, remaining challenges, and future directions

Over the past decades, numerous academic and industry-sponsored clinical trials worldwide have tested various beta cell replacement strategies for the treatment of T1D. Below, we critically evaluate the global landscape of these trials (ongoing, completed, and terminated) with an overview of all known clinical trials to date in this domain, including those using transplanted islet cells (from donors or derived from stem cells), xenogeneic (animal) islets, supportive cell therapies (like T_regs_ or mesenchymal stem cells), and biomaterial implants (encapsulation devices or scaffolds) (**Table 3**). The table details the title, sponsor, therapy type, clinical phase, status, location, key outcomes, and an identifier (an NCT number, if available) for each trial.

**Table 3:** Summary of Clinical Trials for Cell- and Material-Based T1D Therapies.

Trial Title	Lead	Therapy Type	Phase	Status	Region	Key Outcomes	Trial ID
Allogeneic Islet Cell Transplantation in T1D (Lantidra)	NIH (CIT Consortium)/CellTrans	Donor islet infusion (immunosuppression)	3	Completed	USA Canada EU	FDA approved Lantidra June 2023; ~90 % elimination of severe hypoglycemia and ~70 % insulin independence at 1 year (30 % at 2 years).	CIT-07 (NCT N/A)
Autologous Hematopoietic Stem Cell Transplantation (Immune Reset)	Univ. of São Paulo (Brazil)	High-dose immunosuppression (cyclophosphamide) followed by autologous bone marrow stem cell transplant	1	Completed	Brazil	In a small pilot study, intense immune reset led to prolonged insulin independence in the majority of recent-onset T1D patients (168). C-peptide production increased in all but one patient, with some patients remaining insulin-free for years. However, treatment carries significant risks (e.g. infection, chemotherapy side effects), limiting its broad applicability.	NCT00315133
Beta-O2 ßAir Bio-Artificial Pancreas – Oxygenated Islet Macrodevice	Beta-O2 (Israel/Sweden)	Macroencapsulation device with oxygen supply, loaded with human donor islets (retrievable implant)	1	Completed	Sweden	Device implantation was safe with no immune sensitization; encapsulated islets remained viable *in vivo* (16). However, insulin output was minimal (only trace C-peptide) with no impact on glycemic control. Foreign-body fibrotic overgrowth was observed, limiting efficacy.	NCT02064309
CRISPR TX CTX211 – Gene−Edited Islet Therapy	CRISPR Therapeutics (Switzerland/USA)	HIP gene−edited islets in device (no immunosuppression)	1/2	Ongoing	Canada	First−in−human study evaluating safety and insulin production; results expected 2025.	NCT05565248
Encellin ENC−201−CED – Donor Islets in ENCRT Device	Encellin (USA/Canada)	Donor islets in proprietary subcutaneous encapsulation device	1	Ongoing	Canada	First−in−human study: safety of ENCRT device + donor islets.	NCT06408311
LCT “Diabecell” – Encapsulated Porcine Islet Xenotransplant	Living Cell Technologies (NZ)/Otsuka (JP)	Alginate-microencapsulated neonatal pig islet cells (xenogeneic)	1/2	Completed	New Zealand Argentina Russia	Phase I/IIa trials (2009–2014) showed the approach was safe without immunosuppression and led to improved glucose control and reduced insulin requirements in some T1D patients (169). Efficacy was variable and not curative. No zoonoses or serious reactions observed. Further Phase I/IIa trials are planned in 2024 under Otsuka (Japan) to test refined protocols.	(NCT N/A, NZ clinical trial)
Mesenchymal Stem Cell (MSC) Therapy – Uppsala	Uppsala Univ. (Sweden)	Autologous mesenchymal stromal cells (MSC) IV infusion in new-onset T1D	1	Completed	Sweden	Safety confirmed, some preservation of endogenous insulin. C-peptide responses to meal increased post-MSC infusion (170), suggesting improved beta cell function. Effect was transient and sample size was small (n=10).	NCT01068951
MSC + Vitamin D – Rio de Janeiro	Federal Univ. Rio de Janeiro (Brazil)	Allogeneic adipose-derived MSC infusions + daily Vitamin D in new-onset T1D	2	Completed	Brazil	Safety confirmed; patients receiving MSC + VitD showed higher fasting C-peptide levels stable over 6 months (171). Immunomodulatory effects noted, but no patients achieved insulin independence.	NCT03920397
Otsuka OPF−310 – Encapsulated Porcine Islets	Otsuka Pharmaceutical (Japan/USA)	Encapsulated neonatal pig islets (xenotransplant) in device	1/2	Ongoing	USA	Evaluating HbA1c <7% and no severe hypoglycemia; potential off−the−shelf xenograft.	NCT06575426
Polyclonal Treg Therapy (“T-Rex” Study) – Autologous Tregs	Caladrius (Sanford Project, USA)	Autologous T_regs_ infusion (polyclonal Tregs)	2	Completed	USA	Therapy was safe and well-tolerated but showed only modest slowing of C-peptide decline. No significant long-term preservation of beta cell function vs placebo (172). Efficacy was insufficient, and further development of polyclonal Tregs was discontinued. (This Phase II trial, in new-onset T1D adolescents, did not meet its primary efficacy endpoint.)	NCT02691247
Sana UP421 – Hypoimmune Donor Islets	Sana Biotechnology (USA/Sweden)	Gene−edited donor islet cells (hypoimmune) implanted in muscle (no immunosuppression)	1	Ongoing	Sweden	First recipient showed endogenous C−peptide production without immunosuppression.	NCT06239636
Seraxis SR−02 – Manufactured Islet Therapy	Seraxis (USA)	Stem−cell–derived islets implanted on omentum (requires immunosuppression)	1/2	Ongoing	USA	Assessing safety and C−peptide; gene−edited SR−03 trial planned 2026.	NCT06651515
Sernova Cell Pouch™ – Subcutaneous Islet Scaffold	Sernova Corp. (Canada)	Implantable polymer pouch seeded with donor islets (vascularized scaffold; immunosuppression required)	1/2	Ongoing	Canada USA	Phase 1/2 interim: 5/6 patients insulin−independent for 1–5+ years; higher−capacity Cohort B enrolling; manufactured−islet trial planned post−2025.	NCT03513939
Tegoprubart + Donor Islets	Eledon Pharmaceuticals (USA)	Anti−CD40L monoclonal + donor islet infusion	1/2	Ongoing	USA	2 of first 3 participants insulin−independent; milder side effects vs standard regimens.	NCT06305286
Vertex VX-264 – Encapsulated SC-islets	Vertex Pharmaceuticals (USA)	SC-islet cells encapsulated in immunoprotective device (implant)	1/2	Discontinued	Canada USA	Program discontinued in 2025 after initial trial failed to achieve sufficient C−peptide and glycemic endpoints despite safety and tolerability.	NCT05791201
Vertex VX-880 – SC-islets infusion	Vertex Pharmaceuticals (USA)	SC-beta cell replacement therapy (no device; systemic immunosuppression)	1/2/3	Ongoing	USA Canada UK EU	11 of 12 participants in earlier phases reduced or eliminated external insulin; phase 1/2/3 pivotal trial underway; regulatory submission expected 2026	NCT04786262
ViaCyte Enhanced Encap. Device (second-gen PEC-Encap)	ViaCyte/W.L. Gore (USA)	hESC-derived pancreatic cells in improved encapsulation device	1/2	Terminated	USA Canada	Trial halted due to *insufficient functional engraftment* of cell product (173). Device modifications (e.g. Gore membrane) did not overcome host fibrotic response enough to ensure graft function.	NCT04678557
ViaCyte VC-01 (PEC-Encap) – Subcutaneous Encaptra device	ViaCyte (USA)	hESC-derived pancreatic progenitors in encapsulation device (macroencapsulation)	1/2	Completed	USA Canada	Device safety demonstrated but foreign-body fibrosis impeded vascularization; grafts had *no* detectable insulin/C-peptide output (174, 175). Proof-of-concept but insufficient efficacy.	NCT02239354
ViaCyte VC-02 (PEC-Direct) – open vascularized device	ViaCyte (USA) + CRISPR Therapeutics (USA)	hESC-derived pancreatic progenitors in vascularized device (requires immunosuppression)	1/2	Completed	Canada USA	~63% of patients showed successful cell engraftment with insulin-positive cells; ~35% achieved measurable C-peptide by 6 months post-transplant (22). Partial graft function demonstrated (“proof of concept” insulin production).	NCT03163511

Allogeneic islet transplantation has proven that replacing beta cells can restore glycemic control in T1D. The NIH−funded Clinical Islet Transplantation (CIT) trial showed that islet grafts can achieve tight glycemic targets without severe hypoglycemia in patients with unstable T1D (176). In the pivotal Phase 3 CIT−07 study, ~70 % of transplanted patients became insulin−independent for a time, and nearly all were protected from life−threatening hypoglycemia (177, 178). Although insulin independence declined to ~30 % by 2–3 years post−transplant, long−term data confirm sustained graft function and markedly improved quality of life. These results paved the way for the first−ever FDA approval of an islet−cell therapy, donor−islet product Lantidra in 2023. However, broader use is constrained by donor shortage and the need for lifelong immunosuppression.

**Figure 7:**
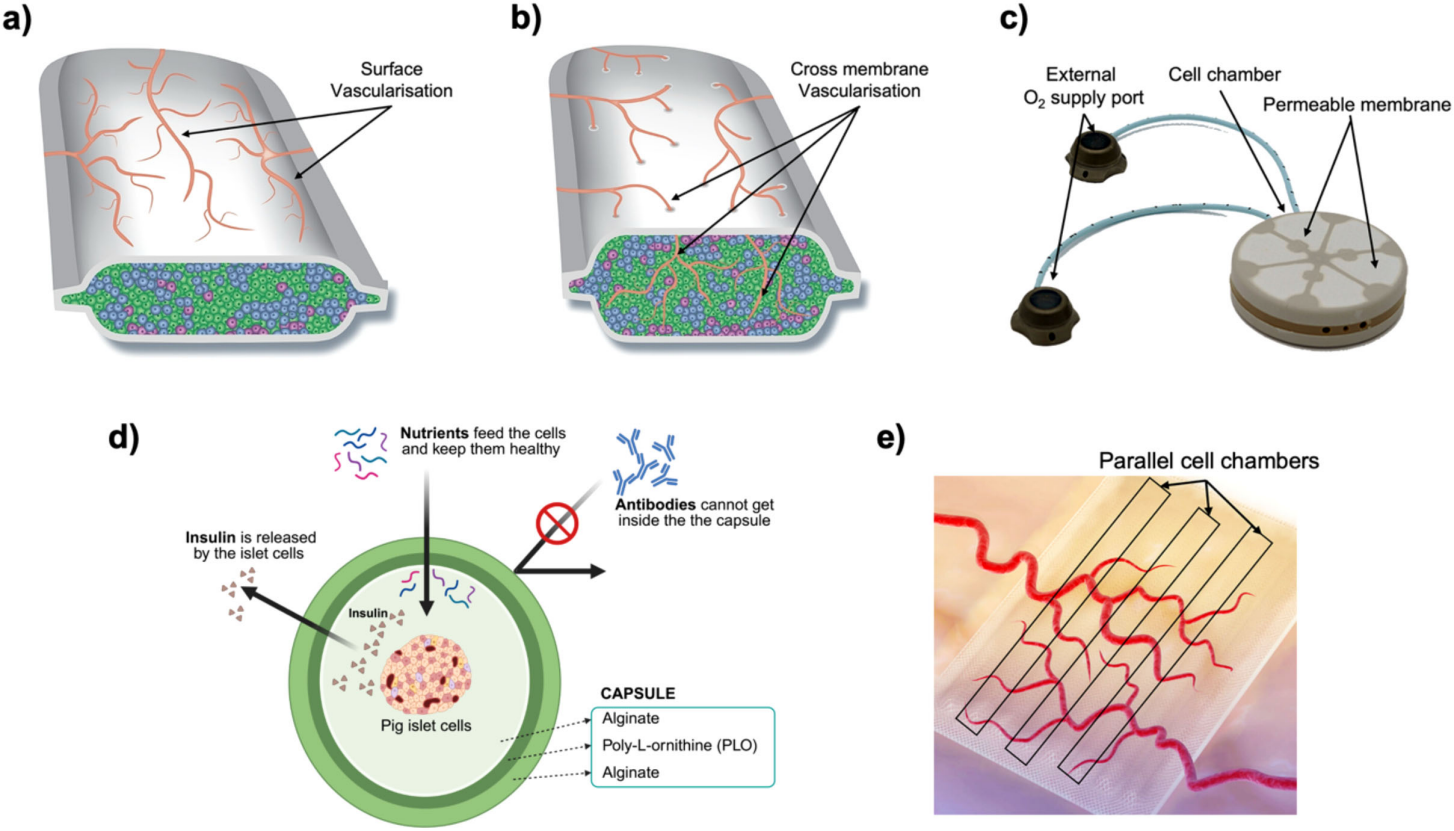
Clinical−stage encapsulation technologies for beta cell replacement therapy. **(a)** ViaCyte Encaptra™ (VC−01): a planar macroencapsulation device comprising parallel, immunoisolating cell chambers sealed within a semipermeable membrane to support subcutaneous implantation and retrievability; evaluated in NCT02239354. **(b)** PEC−Direct™ (VC−02, ViaCyte + CRISPR Therapeutics): an immunoprotective macroencapsulation cassette with a highly permeable membrane allowing direct vascular ingrowth (cross−membrane vascularization) into the islet−filled chambers; currently evaluated in NCT04678557. **(c)** βAir™ (Beta O_2_ + Novo Nordisk): a macroencapsulation chamber fitted with an external refillable oxygen port and oxygen−permeable membrane, supplying supplemental O_2_ to encapsulated islets; assessed in NCT02064309. **(d)** Diabecell™ (Living Cell Technologies): alginate–poly−L−ornithine–alginate microcapsules containing porcine islets, permitting diffusion of nutrients and insulin while excluding host antibodies and immune cells; tested in multiple clinical studies (NCT N/A). Created in https://BioRender.com **(e)** Cell Pouch™ (NVIP−01, Sernova Corp.): a modular, implantable precision−engineered device pre−implanted to establish a vascularized tissue bed; pancreatic islets are subsequently injected into discrete chambers that become perfused by newly formed surface vessels, assessed in NCT03513939.

Pluripotent stem cells offer an unlimited beta−cell source, addressing the donor shortage. ViaCyte pioneered this area with trials implanting pancreatic progenitor cells (PEC−01) derived from hESCs. In their encapsulated product VC−01 (Encaptra), cells survived implantation but the FBR formed fibrous capsules that prevented vascularization, so no meaningful insulin production was detected in patients (**Figure 7a**) (174, 175). Switching to a vascularized device allowing host blood vessels to perfuse the graft VC−02 (PEC−Direct) yielded better outcomes: in an open−label study, 3 of 10 patients maintained clinically significant C−peptide levels (0.1–0.2 nmol L^-^¹) at 1 year—still below physiological levels, but a key proof−of−concept that stem−cell grafts can function in T1D patients (**Figure 7b**) (22).

Vertex’s VX−880 (Zimislecel), now in an expanded Phase 1/2/3 programme, infuses large numbers of stem−cell−derived islet−like cells into the hepatic portal vein under immunosuppression. Remarkably, 11 of 12 participants in the first study cohort achieved ≥ 70 % reductions in exogenous insulin with near−normoglycemic profiles within 90 days (179). These findings underscore the potential of stem−cell−derived beta cells, albeit at the cost of systemic immunosuppression. Vertex also pursued an encapsulated product VX−264 intended to eliminate immunosuppressive drugs; however, the trial was discontinued in 2025 after early implants produced insufficient C−peptide.

An alternate stem−cell programme, Seraxis SR−02, implants fully differentiated stem−cell−derived islets onto the omentum. The ongoing Phase 1/2 trial will evaluate safety and endogenous C−peptide production, while a second−generation gene−edited construct SR−03 designed to reduce immune recognition is scheduled to enter the clinic in 2026.

Another cutting−edge approach is gene−editing transplanted cells to evade immune attack. ViaCyte/CRISPR’s VCTX210 used CRISPR−edited cells lacking key HLA molecules in an encapsulation device (21). This study demonstrated safety and absence of allo−immune rejection in the absence of systemic immunosuppression, although efficacy data remain limited. Building on this concept, CRISPR Therapeutics’ CTX211 is enrolling patients in a Phase 1/2 trial testing hypo−immune stem−cell islets devoid of HLA class I and II within a vascularizing device; initial read−outs are expected in late 2025.

Most strikingly, Sana Biotechnology’s UP421 delivered the first clinical evidence that hypo−immune engineering can work without a protective device: intramuscular implantation of donor islets modified with the HIP platform elicited endogenous C−peptide production in the inaugural recipient without any immunosuppression. Longer−term follow−up and additional patients will clarify durability, but this result points to the possibility of a truly off−the−shelf, drug−free beta−cell therapy.

Biomaterial−based devices aim to protect transplanted cells from immune attack while permitting nutrient and oxygen transport. ViaCyte’s Encaptra pouch failed to sustain adequate oxygenation once scar tissue developed (173). The Beta−O2 βAir device addressed oxygen limitations by incorporating a refillable oxygen reservoir (**Figure 7c**) (180). In a Phase 1 trial, patients periodically injected oxygen into the implanted device (16). This maintained islet viability and prevented immune sensitization, but insulin secretion remained only at trace levels. Upon device retrieval, islets were intact but showed evidence of stress (e.g. amyloid deposition) and blunted glucose responsiveness. Basically, the device kept cells alive but not thriving.

Encapsulation of xenogeneic islets (from pigs) has also been explored. Living Cell Technologies’ microcapsules with neonatal pig islets (Diabecell) were implanted in T1D patients without immunosuppression (**Figure 7d**). These alginate microcapsules avoided acute rejection and were safe over years (169). Partial efficacy was noted such as some patients reduced insulin doses and improved glycemic control modestly, but immune reactions (pericapsular fibrosis) still limited long-term function, and no patients were cured outright. As a result, interest shifted to more advanced biomaterials (e.g. proprietary alginate blends, hydrogels, or devices with immune-modulating coatings) to reduce fibrosis and allow better diffusion.

A different biomaterial approach is Sernova’s Cell Pouch, a porous polymer scaffold implanted under the skin (**Figure 7e**). The pouch is first implanted empty to allow tissue integration and angiogenesis; a few weeks later, donor islets are transplanted into the pre-vascularized chambers (181). Because the islets integrate with the host’s blood vessels, they can function similarly to islets in the liver, so patients do require immunosuppressive therapy. However, the pouch is retrievable and creates a dedicated “organ-like” site for islets in the skin. Interim results are striking: the majority of treated patients became insulin-independent for sustained periods. If confirmed in larger trials, this scaffold approach could offer a safer alternative to intraportal islet infusion (which can involve issues like immediate blood-mediated inflammatory reactions and hepatic complications). The Cell Pouch might also be adaptable to stem cell-derived cells in the future.

Encellin’s ENC−201−CED, a wafer−thin conformal pouch designed for subcutaneous placement, entered first−in−human testing in 2025 to assess safety with donor islets. Concurrently, Otsuka’s OPF−310 couples neonatal porcine islets with a next−generation alginate micro−encapsulation chemistry and has begun a U.S. Phase 1/2 xenotransplant trial, signaling renewed interest in xenogeneic beta−cell sources paired with improved biomaterials.

To reduce the toxicity of conventional calcineurin/mTOR regimens, several adjunct immunomodulatory strategies are in early clinical testing. One example is Tegoprubart, an anti−CD40L monoclonal antibody, administered with donor−islet infusion. In the first three recipients, Tegoprubart enabled two patients to discontinue insulin while exhibiting a favorable safety profile relative to historical immunosuppression protocols.

Beyond replacing beta cells, several trials have targeted the autoimmune process. T_regs_ therapy is designed to suppress autoimmunity and protect residual or transplanted beta cells. Early-phase trials expanded patients’ own T_regs_ ex vivo and reinfused them. These infusions proved safe, without causing generalized immunosuppression or major side effects (172). There were hints of preserved C-peptide and slower disease progression in some individuals, but overall efficacy was limited and did not meet trial endpoints. For example, a Phase II placebo-controlled T_regs_ trial in new-onset T1D (the Sanford Project T-Rex study) found no statistically significant benefit in beta cell function at one year. It became clear that polyclonal Tregs (which are not targeted specifically to islet autoantigens) may not strongly counteract the chronic autoimmune attack in T1D. This has prompted development of next-generation antigen-specific or engineered Tregs (e.g. with chimeric antigen receptors or TCRs for islet proteins), now in preclinical or early clinical stages, but those are not yet in advanced trials for T1D.

Another immune-focused cell therapy involves mesenchymal stem/stromal cells (MSCs). MSCs have immunomodulatory and anti-inflammatory properties and have been tested in T1D to preserve remaining beta cells in new-onset patients. Small trials in Sweden, Brazil, and others have consistently shown that MSC infusions are safe. Some trials reported improved C-peptide levels and lower HbA1c over 6–12 months (170, 171), suggesting a transient preservation of endogenous insulin production. For instance, in one study, MSC-treated patients had higher fasting C-peptide for 6 months than controls. However, the benefits tended to wane over time, and no trial achieved insulin independence with MSC therapy alone. The heterogeneity of protocols (different sources of MSCs, dosing, timing relative to diagnosis) makes it hard to compare results, and larger controlled trials are needed to conclusively determine efficacy. Overall, MSC and Treg trials highlight the difficulty of reining in autoimmunity, a critical piece of the cure puzzle, without long-term systemic immunosuppression.

Another approach attempted in a few academic centers is autologous hematopoietic stem cell transplantation (HSCT) to “reboot” the immune system. In a landmark Brazilian trial, young adults with new-onset T1D underwent high-dose immunosuppressive chemotherapy (to eradicate autoimmune cells) followed by reinfusion of their own blood stem cells. The outcome was remarkable: most patients achieved insulin independence, some for several years, with restoration of endogenous insulin secretion (168). A follow-up of 65 patients found that about half remained insulin-free at 5 years post-transplant, although many eventually relapsed and resumed insulin at lower doses. This suggests that autoimmunity can be put into prolonged remission in some cases. However, HSCT carries substantial risks (e.g. infection, infertility, secondary autoimmune or malignancy risks) and thus is not a routine option for T1D. It does, however, prove the principle that resetting or modulating the immune system can change the course of T1D, especially if done very early in the disease.

The translation of gene-engineered and biomaterial-encapsulated β-cell therapies into the clinic necessitates rigorous ethical oversight and regulatory compliance. At the international level, foundational principles such as those enshrined in the Declaration of Helsinki and the ISSCR Guidelines for Stem Cell Research and Clinical Translation mandate robust informed consent, risk–benefit assessment, and ongoing post-trial safety monitoring. Regionally, the U.S. Food and Drug Administration (FDA) regulates human gene therapy products under the 21 CFR Part 1271 and guidance documents on genome editing, while the European Medicines Agency (EMA) classifies such interventions as Advanced Therapy Medicinal Products (ATMPs), requiring compliance with Regulation (EC) No 1394/2007 and associated Good Manufacturing Practice (GMP) standards. In Asia-Pacific countries, including Japan’s Pharmaceuticals and Medical Devices Agency and Australia’s TGA, tailored frameworks are emerging to accommodate novel ATMPs.

Ethical concerns around irreversible genomic modifications (e.g., CRISPR/Cas9 knockouts) and long-term biomaterial residency demand transparent communication with patients and communities, as well as multidisciplinary review by Institutional Review Boards (IRBs), national ethics committees, and public advisory panels. To navigate these complexities, we advocate for early and sustained engagement with regulatory authorities—through pre-IND/IMPD consultations, adaptive trial designs, and real-world evidence collection—to harmonize safety and efficacy standards across jurisdictions. By embedding such ethical and regulatory foresight into clinical development plans, the β-cell replacement field can responsibly accelerate toward global accessibility and equitable patient benefit.

In summary, early‐phase clinical trials of encapsulated islets and CRISPR-edited grafts have demonstrated safety and preliminary efficacy up to 6–12 months, yet no published studies report outcomes beyond one year nor multicenter, longitudinal trials have assessed durability across diverse patient populations. This temporal gap constrains our understanding of long-term graft function, late-onset immune or biomaterial-related complications, and real-world applicability. However, each setback, whether an encapsulation device failure or only partial cell-therapy success, has yielded invaluable lessons, spurring innovations in synergistic approaches that combine cell-based cures with immune modulation and smart materials. As ongoing Phase II/III studies mature, extended follow-up data will undoubtedly emerge. To ensure these advances translate into a practical, scalable cure for T1D, we urge the field to design and implement 3–5-year, multicenter clinical trials with standardized endpoints such as sustained C-peptide production, comprehensive immunogenicity profiling, and *in vivo* material integrity assessments. Patient‐to‐patient variability, driven by HLA genotype, autoimmune status, age, and environmental exposures, poses a significant challenge to universal β-cell immune escape strategies. Moving forward, clinical trials should incorporate pre-transplant genomic and immunologic profiling, including high-resolution HLA haplotyping to inform donor–recipient matching (182), and comprehensive islet autoantibody panels to gauge baseline autoreactivity (183). Single-cell transcriptomics and spatial multi-omic profiling of peripheral blood and graft biopsies can further delineate immune activation states and identify cell-type–specific signatures predictive of graft acceptance or rejection (184). Cytokine and soluble biomarker panels, analyzed via high-throughput multiplex assays, may reveal systemic inflammatory milieus that correlate with early graft outcomes (185). Finally, integrating these multi-omic datasets with machine-learning algorithms such as microRNA-based disease risk scores, can enable the development of predictive biomarkers and adaptive trial designs that tailor immunomodulatory regimens to individual risk profiles. By embedding these precision-medicine approaches into longitudinal, multicenter studies, the field can optimize the efficacy and safety of β-cell replacement across genetically and immunologically diverse patient populations. Though the path has been challenging, the convergence of recent scientific breakthroughs makes the goal of a durable, cell-based cure more promising than ever before.
